# Characterisation of pH variations along the Ba River in Fiji utilising the GEF R2R framework during the 2019 sugarcane season

**DOI:** 10.1007/s10661-021-09423-1

**Published:** 2021-11-19

**Authors:** Nicholas Metherall, Elisabeth Holland, Sara Beavis, Adi Mere Dralolo Vinaka

**Affiliations:** 1 Pacific Centre for Environment and Sustainable Development, University of the South Pacific, Suva, Central Division Fiji; 2 Australian National University - Fenner School of Environment and Society, Canberra, ACT Australia; 3 The University of the South Pacific, Suva, Central Division Fiji

**Keywords:** Ridge-to-reef (R2R), Rapid sampling, Water quality, Pacific Small Island Developing States (SIDS), Catchment disturbance, Coastal ocean acidification

## Abstract

Within Pacific Small Island Developing States (Pacific SIDS), the ridge-to-reef (R2R) approach has emerged as a framework for monitoring river connectivity between terrestrial and marine ecosystems. The study measured water quality, including pH, over 88.40 km of the Ba River in Fiji. The sampling design focused on measuring spatio-temporal variability in pH throughout the sugarcane season with three rapid sampling periods (RSP1, 2 & 3) along the Ba River, together with continuous measurement of temperature and pH using stationary data loggers at two locations upstream and downstream of the sugar mill. Spatial variability in pH and water quality was characterised before (RSP1 and RSP2) and during (RSP3) the sugarcane season. Mean pH measured before the sugarcane crushing season for RSP1 and RSP2 were 8.16 (± 0.49) and 8.20 (± 0.61) respectively. During the sugarcane crushing season (RSP3), mean pH declined by 3.06 units to 6.94 within 42 m downstream of the sugar mill (*P* ≤ 0.001). The 3.06 unit decline in pH for RSP3 exceeded both the mean diurnal variation in pH of 0.39 and mean seasonal variation in pH of 2.01. This decline in pH could be a potential source of acidification to downstream coastal ecosystems with implications for coral reefs, biodiversity and fishery livelihoods.

## Introduction

Environmental monitoring in water management programs has often been singularly focused: either at the catchment scale of the terrestrial realm or at the coast to reef scale of the marine realm. Integrated Water Resource Management (IWRM) programs have expanded across the Asia–Pacific region with a focus on land use change monitoring, forestry and the conservation of soil and water quality to support upland agricultural-ecosystems (Druschke, 2013; FAO, 2005, 2011; Kaiser, 2014; Lamb, 2011; SPREP, 2007; Zeng et al., 2018). Marine conservation programs have often encompassed regulation of fishing activities to support food security, livelihoods or biodiversity outcomes (Moritz et al., 2018; CTI-CFF, 2017; Pietri et al., 2015*;* Haapio et al., 2014; ADB, 2014; Foale et al., 2013; Fidelman et al., 2012; Christie et al., 2011; Veitayaki, 1998). These siloed approaches have often struggled with assessing the complex interplay across the land-sea boundary. Since 1991, with the Global Environment Facility (GEF) pilot phase and subsequent GEF rounds 1–8, the approach has pursued the potential to connect science to more holistic development needs by bringing the IWRM (GEF, 2007; GEF, 2006; GEF, 1999a, 1999b) and Integrated Coastal Management approaches (GEF, 2009; GEF, 2005; GEF, 1999a, 1999b) into a single river catchment framework (GEF, 2018a, 2018b; GEF, 2013). A range of studies have advocated for the development of institutional capacity that focuses on adaptable water quality monitoring of ecological connectivity in rivers extending to reefs and coastal ecosystems (McCauley et al., 2019; Zinabu, et al., 2019; Beavis, 2005). The US Geological Survey’s recent development of an ‘Integrated Water Science Basins’ monitoring framework is one such example (Van Metre et al., 2020). In the Pacific, land-coast-sea scientific frameworks have promoted a ‘ridge-to-reef’ (R2R) approach to take into account the connectivity between the various socio-ecological systems linking islands and oceans (Hilty et al., 2020; Li et al., 2020; von Shuckmann et al., 2020; Baker-Medard, 2019; Carlson et al., 2019; Comeros-Raynal et al., 2019; Bainbridge et al., 2018; Delevaux et al., 2018). By focusing on river corridor waterways through to coasts and reefs (or ocean), the R2R approach provides a framework for monitoring the river and its function in providing ecological connectivity between terrestrial and marine ecosystems.

The GEF R2R environmental monitoring and conservation program implemented at the river catchment level has established 14 country projects across the Pacific (SPC, 2016). The GEF R2R program includes the country scale System for Transparent Allocation of Resources (STAR) initiative (SPC, 2018) as well as the Regional International Waters R2R Project (SPC, 2020). The objectives of the R2R initiative are framed within a diverse range of GEF focal areas including biodiversity, land degradation, climate change adaptation and mitigation, integrated water and sustainable forest management (UNDP, 2020). Environmental monitoring and assessment of the connectivity between terrestrial and marine ecosystems within the R2R framework also align closely with the Sustainable Development Goals (SDGs), specifically targets 14.1 ‘to reduce marine pollution… from land-based activities’ and 15.1 to ensure conservation of ‘terrestrial and inland freshwater ecosystems and their services’ (Holland et al., 2019; SDSN, 2021; UNSDSN, 2020). Program funding for these 14 country pilot projects has depended on GEF budgetary support between 2015 and 2021 (UNDP, 2020). The short timeframes for projects and their funding make long-term environmental monitoring and assessment of complex processes a challenge. Monitoring of the interconnected relationships between landscapes, waterways and coastal ecosystems in Pacific Small Island Developing States (SIDS) requires consideration of complexity (Ourbak & Magnan, 2018; Katafono, 2017). Generating insights into R2R processes and the inputs into global ocean processes can take many years. Currently, most environmental monitoring and assessment frameworks require timelines, budgets, research and data collection resources not yet widely available in Pacific SIDS contexts (notwithstanding the expanding role of local capacity including the University of the South Pacific and its local affiliate organisations). Consequently, strategic and novel experimental designs are needed to address these environmental monitoring and assessment challenges.

**Fig. 1:**
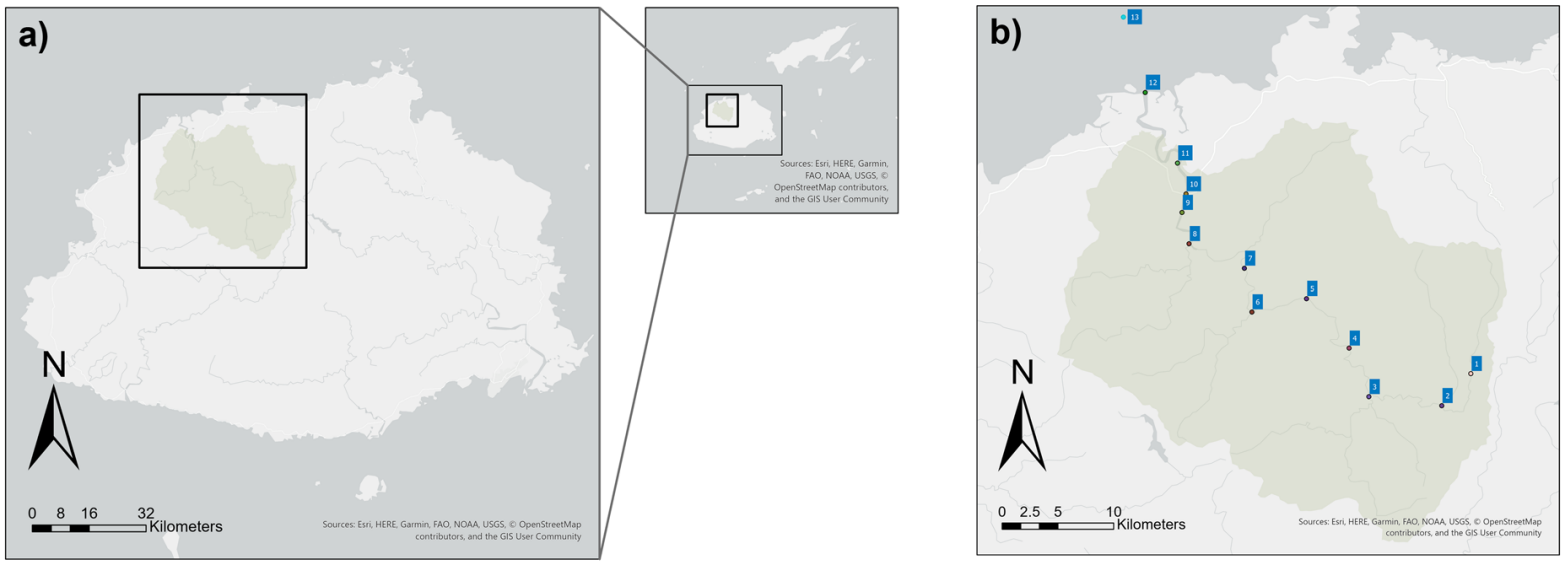
**a** Ba River catchment study area located in Northern Viti Levu. The river catchment boundaries are defined by topographical data. **b** The 13 sub-sections of the 88.40 km length of the Ba River in Viti Levu, Fiji. The Ba Catchment boundaries were generated using a digital elevation model (DEM)

The Fiji national R2R Project has received widespread support through co-financing partnerships amongst the National Government, the GEF and UNDP and technical expertise from conservation NGOs, research institutes and consultancies (SPC, 2016; GEF, 2016; Wilson, 2015). Seven priority catchments were identified for the pilot STAR R2R environmental monitoring projects including four catchments on the main island of Viti Levu: Ba River, Tuva River, Waidina River and Rewa Delta. Further three catchments were identified for the second largest island of Vanua Levu including Labasa River, Vunivia River and Tunuloa district. This study focuses on the Ba River catchment shown in Fig. 1a. The catchment falls within Ba District, one of four districts of Ba province, the most populous province in Fiji with 247,708 residents making up 28% of the national population, as identified by the 2017 Census (Fiji Bureau of Statistics, 2018). The Ba catchment provides a rural agrarian case study representative of the Western Division ‘sugarcane belt’ provinces of Fiji. A range of studies have linked agricultural runoff from sugarcane crops to impacts on waterways and coastal water quality (Yu et al., 2018; Nhiwatiwa et al., 2017; Wooldridge, 2009; Kwong et al., 2002). Sugarcane is the primary industry of Ba District. One of the most important agricultural crops planted in Fiji, sugarcane, has been an essential primary agricultural industry and source of exports for Fiji’s economy for most of the nineteenth and twentieth centuries (Naidu et al., 2017). According to the Observatory of Economic Complexity (OEC) World Trade Database, Fiji is categorised as ‘highly specialised’ in production and export of sugar or ‘sugar preserved foods’ (OEC, 2020). More specifically, Fiji’s agricultural sector has come to specialise in the *Mana* variety of sugarcane (*Saccharum* spp.), which is a mid-late season maturing variety that contributes approximately 65% of national cane production (Naidu et al., 2017). In the 22 years between 1996 and 2018, both the value and quantities of exports (tonnes) have experienced a steady decline with an 82.46% decline in value and a proportional 83.06% decline in quantity (FAOSTAT, 2018). In 2018, Fiji’s raw sugar exports made up only 37.74 million USD or 3.68% of the total value of all national exports (OEC, 2020; FAO, 2020). Through the 2018–2019 season, the Fiji Sugar Corporation (FSC) Rarawai mill crushed a total of 487,483 tonnes of sugarcane associated with point and nonpoint discharge pollution into the Ba River (FSC, 2020a, 2020b). As a result, this study seeks to measure water quality variations in the Ba River throughout the 2018–2019 sugarcane season.

Water quality monitoring provides a tool for rapid sampling and assessment of both point and nonpoint sources of environmental contaminants into waterways. Water quality data can also identify sources and pathways of potable water, ecological connectivity and nutrient inputs. Experimental sampling can be used to detect contaminants and anthropogenic pollution exchange between the land and the ocean. The extent to which these water quality sampling strategies can be improved either temporally by optimizing sampling frequency, or spatially by optimizing the number of locations for sampling is often limited by budget implications and time (Destandau & Zaiter, 2020). Ideally, water quality sampling should be coupled with ecological sampling that measures the range, richness and spatial distribution of species assemblage (Green & Vascotto, 1978; Turner & Trexler, 1997; Stewart-oaten, 1996). As with water quality sampling, the cost of extensive replication of ecological sampling limits broad-scale monitoring of trends in aquatic invertebrate biodiversity (Halse et al., 2002). Both the Pacific regional and national level R2R programs incorporate collection of water quality and ecological sampling into their environmental assessment reporting including Rapid Coastal Assessment (RapCA), Rapid Resource Assessment (RRA) and diagnostic analyses reports. However, the short timelines of these environmental assessments limit the ability to capture temporal variability of water quality.

Various water quality parameters were sampled in this study although pH became a particular focus suited to monitoring potential contributions to coastal ocean acidification. As a result, this study adds to a growing body of case studies sampling water quality to assess priority areas in relation to SDG target 14.3 which seeks to ‘minimize and address the impacts of ocean acidification, including through enhanced scientific cooperation at all levels’ (SDSN, 2021). While acidification in the open ocean is more commonly attributed to anthropogenic emissions, acidification of coastal waters is often causally linked to catchment processes (Aufdenkampe et al., 2011; Duarte et al., 2013). The influence of these catchment processes including runoff, sediment and nutrient cycles on alkalinity and CO_2_ fluxes, for example, can lead to decadal changes of up to 0.5 units in coastal ocean pH (Duarte et al., 2013:221). Furthermore, the spillover effects of increasing ocean acidification, retrograde solubility of C in the ocean and the thermal inertia of the ocean all contribute to the much longer timescales of climate change (Soldatenko & Yusupov, 2019; Abdusammatov et al., 2012; Manabe et al., 1990). There is a resulting disjoint between longer-term timelines of local coastal and global ocean processes reinforced by water quality sampling and short-term environmental monitoring project timelines and budgets. Within rivers, pH is affected by a range of biological and physicochemical processes (Tibby et al., 2003). In aquatic ecosystems, photosynthesis produces oxygen and raises pH; aerobic respiration consumes oxygen and lowers pH (Hamid et al., 2020). These processes are, in turn, influenced by temporal variability in temperature, CO_2_ fluctuations and acidic inputs from rainfall and soils (Hamid et al., 2020). The magnitude and frequency of temporal fluxes in water pH varies by ecosystem, geography and depth of sampling in the water column. For example, pH fluctuations measured in the open ocean range from 8.06 to 8.10 over a mean 30-day period (Duarte et al., 2013; Hofmann et al., 2011). Fluxes in coastal ocean surface water pH sampled around the Great Barrier Reef, Australia, ranged more widely from 7.69 to 8.30 on a diurnal basis (Santos et al., 2011). In contrast to oceans, the water quality within rivers is more immediately exposed to both anthropogenic and natural catchment processes. To support ecosystems, pH in rivers ranges between 6.50 and 8.50 (Dodds, 2006; Chapman, 1996). A range of landscape processes contribute inputs of acidity and alkalinity. For example, inputs such as acid mine drainage, acid-sulfate soil runoff and acid rain all decrease pH (Beavis et al., 2005, 2006). Extremes in pH can make a river inhospitable to aquatic biota. Acidic water leads to increased chemical weathering, disturbance and leaching of heavy metals harmful to ichthyofauna and benthic populations (Beavis, et al., 2006). Acidic inputs can be buffered by alkaline inputs such as carbonate ions mobilised into waterways through chemical and physical weathering of limestone, and by ingress of seawater in estuaries. Riparian vegetation functions as an additional natural buffer, filtering runoff into streams to maintain water quality and biogeochemistry equilibria (Kuglerová et al., 2014; Hawes & Smith, 2005; Mander et al., 2005). Efficient riparian buffer widths range from approximately 3 m for bank stabilization and stream shading, to over 300 m to provide habitats for wildlife (Hawes & Smith, 2005). Guidelines for environmental monitoring of estuaries (ANZECC & ARMCANZ, 2000) recommend that assessment should consider various modes of impact such as triggers, multiple stressors, cumulative effects and thresholds. The concept of ‘indefinite resilience’ raised by Kelly et al., (2014) refers to the ability of an environment to ‘absorb a given amount of a stressor in perpetuity, rather than in a time-bound capacity’. It is evident from previous project outputs and from the literature that Fiji’s catchments operate in a multi-stressor environment as shown in the country project design (UNDP, 2020).

The goal of this study is to apply a rapid sampling design strategy to document the temporal and spatial dynamics of pH from the ridge of the Ba River to the downstream reef. The study aims to document a single sugarcane season with a focus on the harvest to crushing portion of the season because of the potential impact on water quality. The rapid sampling strategy was designed to inform the Pacific regional rapid assessment or the GEF R2R framework using Fiji as a case study. The study design utilised the ridge-to-reef framework as its spatial dimension and focused on two temporal dimensions including characterising the diurnal cycle of pH and the impact of the sugar cane crushing season. A surface water quality assessment was conducted from ridge to reef along 88.40 km of the Ba River in the Republic of Fiji, hereafter referred to as Fiji. The study explores variability in surface-water pH in the context of the indefinite resilience of the Ba River ecosystems’ ability to absorb a range of disturbances (ANZECC & ARMCANZ, 2000). In Fiji, surface and ground water quality data are not collected systematically, nor is the data organised for accessibility (FAO, 2016a, 2016b). Rural and remote areas of Fiji, in particular, face combined challenges relating to constrained access to information and resources, and limited capacity for water quality monitoring (Kirschke et al., 2020). The limited long-term water quality monitoring datasets for Fiji’s rivers are managed by the Ministry of Environment and Waterways (MoEW). Ba River catchment is a data-limited context. To address this gap, this study sampled pH along the Ba River through a process of rapid spatio-temporal sampling to identify anomalies in pH. Geotagged water quality anomalies provide inputs into a baseline of environmental monitoring and identifiy the location of point and diffuse source pollution hotspots.

## Materials and methods

### Study area description

The Ba River drains a catchment area of approximately 932 km^2^ equivalent to 8.97% of the total landmass of Viti Levu. As part of the lee-ward northwest region of Viti Levu, Ba Province experiences a greater variability of mean annual precipitation and a greater likelihood of drought, due to orographic rain shadow effects (Kumar, 2010; Mataki et al., 2006). Within this study, the Ba River catchment area was divided into 13 river segments. These segments were delineated in a geo-database using criteria of elevation, slope, geology, vegetation, land use, tributaries, fresh and marine water resources and hydrological characteristics. Each of the segments was measured in terms of distance downstream of sampling point 1 (SP1) near the ridge headwaters recorded at S17.609320° E177.933518°. The upper catchment, extending from SP1 at 0.00 to 44.00 km downstream along the length of the Ba River, was divided into six discrete segments (1–6) as outlined in Table 1. The lower catchment and floodplain extending from 44.00 km to sea level elevation at 73.00 km (from SP1) was divided into five discrete segments (7–11) as shown in Table 2. The marine zone is composed of two segments (12–13) including the extensive area of mangrove estuarine delta, and the coastal ocean extending out towards the reef at 88.40 km (from SP1), also described in Table 2. All distances marked along the Ba River R2R sampling line refer to longitudinal distances downstream of SP1. The 13 segments (Fig. 1b) were delineated using datasets from Landsat 8 and Sentinel 2 satellite Earth observation imagery (Earth Engine, 2020; USGS, 2020), geological maps (Rodda, 1966; Lagabrielle et al., 1994) and hydrogeological maps (Gale, 1991). To verify field observations of the catchment, ecological datasets were also used, including riparian vegetation, flora and mangrove species (Veitayaki et al., 2017; Tuiwawa et al., 2013; FAO, 2011; Ellison and Fiu, 2010; Taba et al., 2005) and fauna of the aquatic and marine biota (Rashni, 2020; Paris et al., 2019; Veirus et al., 2018; Hewavitharane et al., 2018; Ledua et al., 1996). Hydrology and catchment management reports also provided additional information (FAO, 2011).

**Table 1:** Description of the Ba River catchment from SP1 to Toge Village including ridge-to-reef segments 1–6 (0.00–44.00 km from SP1)

Ecosystem reach type and distance	Segment 1. Headwater to Nadarivatu hydropower (0.0–9.34 km)	Segment 2. Nadarivatu hydropower to Savatu confluence (9.34–15.5 km)	Segment 3. Savatu confluence to Nakara confluence (15.5–24.5 km)	Segment 4. Nakara confluence to Navala Village (24.5–31.8 km)	Segment 5. Navala village to Koroimavua andesite (31.8–40.2 km)	Segment 6. Edge of Koroimavua group to Toge Village (40.2–44.0 km)
Relief mean elevation (m) [min, max]	424 [245, 603]	294 [151, 247]	119 [81, 166]	69 [53, 86]	49 [25, 72]	27 [16, 52]
- gradient: mean, max (%)	− 3.8, − 15.1	− 2.3, − 6.3	− 2.2, − 8.6	− 1.3, − 4.8	− 1.5, − 4.6	− 1.7, − 8.6
Soils	Well drained, aerated, udic clay loam. Slightly to moderate acidic, erosion risk potential	Well drained, aerated, udic clay loam. Slightly to moderate acidic, erosion risk potential	Well drained, aerated, udic clay loam. Slightly to moderate acidic, erosion risk potential	Well drained, aerated, udic clay loam. Slightly to moderate acidic, erosion risk potential	Well drained, aerated, udic clay loam. Slightly to moderate acidic, erosion risk potential	Well drained, aerated, udic clay loam. Slightly to moderate acidic, erosion risk potential
Geology	Nadarivatu undifferentiated pliocene basaltic and derived flows: olivine-bearing with minor epiplastics: andesinite and basalt	Inclined bedding at the site of the hydropower station. Nadarivatu undifferentiated pliocene basaltic and derived flows: olivine-bearing with minor epiplastics: andesinite and basalt. Fault line marks the end of this segment at Savatu confluence	Fault line at Savatu. Mostly volcanics including Vatukoro group basalt derived sandstone connecting to small pockets of Korombalavu andesite, massive lava and volcanic breccia with limestone	Pliocene Koroimavua andesitic group connecting to Ba volcanics sandstone derived from basalt. Crossing through Nadi and Sigatoka sedimentary groups. A fault line exists at Navala Village	Pliocene Koroimavua andesitic group connecting with Nadi Sedimentary group: undifferentiated conglomerate marl, limestone and andesitic rocks	Pliocene Koroimavua andesitic flows and pyroclastics grading southwestward into sandstone. Connecting to Vatukoro (basalt-dervied) sandstone. Toge Korombalavu andesite, massive lava, volcanic breccia, limestone
Riparian vegetation	20–250 m riparian buffer width made up of native forest. Diverse highland flora connected to 74.06km^2^ P*inus carribaea* pine forest reserves adjacent to Ba River catchment (FAO, 2011)	15–200 m riparian buffer width made up of native forest. Connected to 74.06km^2^ *P. carribaea* pine forest reserves adjacent to Ba River catchment (FAO, 2011)	Orographic effect of rainshadow leading to domination of *talasiga* grasslands with limited forest catchment area including *Calliandra calothyrsus* and Indigenous tree species (FAO, 2011)	Continued *talasiga* grasslands with limited forest catchment area including raintrees (*Samanea saman*) and *C. calothyrsus*	Medium sized forested sub-catchment area including *C. calothyrsus, S. saman* and mango trees (*Mangifera indica*)	Agriculture and small forest sub-catchment area including *C. calothyrsus, S. saman* and *M. indica*
Villages/settlements	Nayacana,	Marou, Buyabuya, Drala, Koro. Hydropower station	NA	Navala Village	Isolated agriculture settlements	Small to medium scale agriculture
Sub-catchments/tributaries (no. of stream order)	-Upper Ba (Nabiaurua) sub-catchment -Naidadara (2)	-Nabiaurua sub-catchment -Multiple 1st-order streams	-Nabiaurua + Nakara sub-catchments -Savatu 2nd-order river	-Nakara, Saqunu, Navuniyasi sub-catchment -Naweni (3), Nakara (4)	-Saggunu, Navuniyasi, Wainimau, sub-catchments -Three 1^st^, 2^nd^ order streams	-Wainimau and Waisali sub-catchments
Freshwater resources	Eels (*Anguilla australis*) and tilapia (*Oreochromis niloticus*), giant prawns (*Macrobrachium rosenbergii*)	*A. australis O. niloticus*, *Macrobrachium. Spp*	*A. australis O. niloticus*	*A. australis O. niloticus*	*A. australis O. niloticus*	*A. australis O. niloticus*
Hydrology - Streambed substrate - reach types	Beginning of the stream providing low turbidity water (0.00–0.50 NTU). Bedrock, huge boulders and cobbles. Pools and riffles	Large boulders, large cobbles, small cobbles, pebbles and silt. Pools, cascades, riffles and shallow runs	Large boulders, large cobbles, small cobbles, pebbles and silt. Pools, large riffles and shallow runs	Boulders, large cobbles, small cobbles, pebbles and silt. Pools, large riffles and runs	Boulders, large cobbles, small cobbles, pebbles and silt. Pools, large riffles and runs	Boulders, large cobbles, small cobbles, pebbles and silt. Pools, small riffles, deep runs

**Table 2:** Description of Ba river catchment floodplains from Toge Village to reef including ridge-to-reef segments 7–13 (44.00–88.40 km)

Ecosystem reach type distance	Segment 7. Toge village to agriculture area (44.0–50.0 km)	Segment 8. Large agricultural area (50.0–57.6 km)	Segment 9. Moto River to Old Ba Bridge (57.6–61.7 km)	Segment 10. Old Ba Bridge to Ba Bridge (Town) (61.7–63.3 km)	Segment 11. Ba Bridge to Mangrove (63.3–73.0 km)	Segment 12. Mangrove extent (73.0–78.0 km)	Segment 13. Mouth of river to reef (approximately 78.0–88.4 km)
Relief- mean elevation (m) [min, max]	14 [8,21]	10 [6, 14]	5 [0, 13]	1 [0, 2]	1 [0, 2]	0 [0, 0]	0 [0, 0]
Gradient: - mean, max (%)	− 0.8, − 2.5	− 0.6, − 1.6	− 0.9, − 3.9	− 0.2, − 0.7	− 0.2, − 0.5	− 0.1, − 0.5	− 0.0, − 0.0
Soils	Well drained, very good aeration. Gravelly clay loams. Ustic and acidic. Significant erosion	Well drained, very good aeration. Gravelly clay loams. Ustic and acidic. Significant erosion	Well drained, very good aeration. Gravelly clay loams. Ustic and acidic. Significant erosion	Well drained, very good aeration. Gravelly clay loams. Ustic and acidic. Significant erosion	Well drained, very good aeration. Gravelly clay loams. Ustic and acidic. Significant erosion	Well drained, very good aeration. Gravelly clay loams. Ustic and acidic. Significant erosion	Alluvial surficial deposits. Well drained, very good aeration. Gravelly clay loams. Ustic and acidic. Significant erosion
Geology	Pleistocene Vatukoro (basalt -derived) sandstone connecting to alluvial sands, silts and muds	Undifferentiated pleistocene, alluvial surficial deposits connected to some areas of volcanic basalt derived sandstone	Undifferentiated pleistocene, alluvial surficial deposits: sands, silts and muds. Connected to some areas of basalt derived sandstone	Undifferentiated pleistocene, alluvial surficial deposits of alluvial sands, silts and muds	Undifferentiated pleistocene, alluvial surficial deposits: alluvial sands, silts and muds	Undifferentiated pleistocene, alluvial surficial deposits. Mud and silt along the banks of Ba River. Soft muddy banks meet hypersaline mudflat estuary streambeds	Estuarine mud banks border the mangroves. Hypersaline mudflats continue out to the ocean to meet the reef composed of carbonate coral structures
Riparian vegetation	Riparian vegetation including *C. calothyrsus, S. saman M*.*indica*)	Thin line of riparian vegetation and shrubs including *C. calothyrsus, S.sam*an and *M.indica*	Large forests including *C. calothyrsus, S.saman* and shrubs. Some areas with sparse lines including *M.indica*	Thin riparian lines of trees. Some areas with sparse lines including *C. calothyrsus, S. Saman, M.indica*	Thin line of riparian vegetation including *S.saman, C. calothyrsus,* and *M.indica*	Mangrove species including *Rhizophora stylosa, Rhizophora mangle, Rhizophora selala* (Ellison & Fiu, 2010)	Algae, micro-algal mats, coral. *R.stylosa, R.mangle, R.selala* (Ellison & Fiu, 2010)
Land use	Medium-scale agriculture: sugarcane, root crop	Extensive large-scale agricultural area with sugarcane and horticulture	Moderate-scale agricultural area and sugarcane on the right bank. Nasolo Village	Ba town with drainage and increased areas of industrial and residential runoff	Extensive sugarcane fields. Nailaga and Votua villages	Downstream of Nawaqarua: the last village along Ba River. Some adjacent sugarcane fields	AMEX mining for iron magnetite. Fishing and transport boats: a source of hydrocarbons
Sub-catchments/tributaries (no. of stream order)	-Waisali, Navisa -Balevuto (5) Waisali (3)	-Navisa, Nadrou sub-catchments	-Floodplain sub-catchment -Moto, Nadrou, Variciva	-Floodplain sub-catchment -Namosau, Veisaru	-Floodplain sub-catchment -Nabawalu	-Floodplain sub-catchment	-Floodplain sub-catchment
Marine resources	*A. australis* and *O. niloticus*	Freshwater mussels (*Batissa violacea*)	*B. violacea* (Ledua et al., 1996)	*B. violacea*	*B. violacea*	Hammerhead (*Sphyrna lewini*), blacktip shark (*Carcharhinus limbatus*)*,*	*S. lewini, C. limbatus*
Hydrology - Streambed substrate - reach types - tidal extent	Small cobbles, pebbles, silt, sands, mud. Pools, large riffles and shallow runs	Small cobbles, pebbles, silt, sands, mud. Pools and runs. Flood zone	Pebbles, silt, sands, mud. Pools and deep runs. Point of tidal influence extent. Flood zone	Freshwater mixing zone: salinity = 0.5–1 PSU. Deep straight run. Flood zone	Mud, silt. Deep runs. Salinity = 1–10 PSU. Flood zone	Mud flats with some sand and silt. Deep runs. Marine influence and flood zone: salinity = 10–20 PSU	Mud flats, basalt sands, towards reefs. Ocean: salinity = 20 + PSU

The upper-catchment segments 1 to 6 begin at the headwater ridges. Spring water flowing into the reaches of Ba River at SP1 was observed at ~ 1060 m elevation. The highland Ba Volcanic groups of the early Pliocene dominate the geomorphology of the river channel (Rodda, 1966; Stephens et al., 2018). At the headwater springs of the Ba River, SP1 recorded low levels of turbidity (0.00 to 0.50 NTU). Riparian vegetation provides thick buffer zones composed of native forest and shrubs ranging in width from approximately 20 to 250 m, up until 6.20 km downstream of SP1 at the first riverside village: *Marou*. The streambed substrate is largely composed of cobbles, boulders and bedrock. The stream itself is characterised by sequences of pools and riffles and then sharp ravines where the river course cascades underneath large limestone boulders and cavernous systems rendering many of these reaches inaccessible. At 9.34 km downstream of SP1, Energy Fiji Limited (formerly Fiji Electricity Authority) generates hydro-electricity through the Nadarivatu Dam by channeling water through a tunnel from the Sigatoka river headwaters into the Ba River (EFL, 2012). Adjacent to the dam and the Ba River is an area of approximately 74.06 km^2^ of *Pinus carrabea* pine forest reserves (Fiji ERP, 2019; FAO, 2011). The remaining upper catchment’s highland vegetation is characteristic of the wider unforested northwestern rainshadow (Yeo et al., 2007*;* Ferese et al., 2000; Zed, 1987).

At 44.00 km, the Ba River meets the lower-catchment floodplain at an elevation of 25 m above sea level. The floodplain is largely composed of agricultural land: primarily sugarcane. Here, surface water from the Ba River is commonly abstracted for irrigation. The width of the riparian vegetation buffer through lower-catchment segments 7 to 11 ranges from 0 to 40 m with agricultural land often extending up to the banks of the river. The FSC Rarawai mill is located on the Ba River right bank at 62.19 km downstream of SP1. Ba Town adjoins the river from 62.30 km to Ba Town Bridge at 63.30 km. Within this reach, extensive sugarcane fields also occur with limited riparian buffer zones composed largely of tropical dry forest (Keppel & Tuiwawa, 2010). Some examples of tropical dry rainforest species observed along the Ba River include raintrees: (*Samanea saman*), mango (*Mangifera indica*), diverse family of Rubiaceae trees and shrubs, (*Leucaena leucocephala*) and african tulip (*Spathodea campanulata*) (Keppel & Tuiwawa, 2010). Agricultural land with limited riparian vegetation continues to line the Ba River until the marine zone at a mean elevation of 0 m (sea level).

The marine zone including segments 12 and 13 commences with the mangrove ecosystem at 73.00 km downstream of SP1 that extends a further 5.00 km to the mouth of Ba River. The Ba estuary has the largest contiguous area of mangrove in Fiji made up of *Rhizophora stylosa, Rhizophora mangle* and *Rhizophora selala* (Ellison and Fiu, 2010). GIS mapping estimates the mangrove area of Ba to reach approximately (50.71 km^2^). The Ba estuary is dominated by mud flats (Paris et al., 2019). Reef was found at a longitudinal distance of 82.40 km and continued beyond the extent of sampling at 88.40 km. The spatial extent of the reef was also verified through the use of open-source geodatabases (Reefbase, 2020; UNEP, 2018; NASA, 2015; NASA, 2009) as well as other Landsat and Sentinel image collections. The status of these marine ecosystems can be gauged through ecological studies using bioindicators. There have been very few studies related to the coral reef fauna of Ba District’s coast and no long-term ecological studies (WWF, 2003; Morris, 2007; Vuki et al., 2000). Marine surveys of fisheries have found planktivore dominant trophic groups of fish with high abundance and low biomass (Tuqiri, 2009). Shark studies have found hammerhead sharks (*Sphyrna lewini or Sphyrna mokarran*), blacktip sharks (*Carcharhinus limbatus*), gray reef sharks (*Carcharhinus amblyrhinchos*), nurse sharks (*Nebrius ferrugineus*), whitetip reef sharks (*Trianedodon melanopterus*) and bull sharks (*Carcharhinus leucas*) (Paris et al., 2019; Veirus et al., 2018).

A land use classification (Yeo et al., 2016) summarised Ba Catchment’s composition as 30.69% natural native forest including 6.12% dense, 12.88% moderately dense and 11.70% scattered forest. Ba Town is composed of the town centre, market and Rarawai FSC mill and residential settlements making up approximately 2.42 km^2^ on the east side of the river and residential settlements and industries making up approximately 7.06 km^2^ on the west of the river comprising a total urban area of approximately 1.02% of the entire catchment. The largest area of the catchment is grasslands cover making up 41.15% of the entire catchment. These ‘*talasiga’* or ‘sun-burnt’ grasslands in Fiji are typical of the drier leeward rainshadow section of Viti Levu (Qamese, pers comm, 2018; Gillison et al.,^2014^). Plantation forest (mostly *Pinus carrabea*) represents 11.05% of the total catchment area. Sugarcane makes up the largest crop type in terms of the area of the agricultural land cover class making up 16.09% of the entire catchment.

The stages of growing, cutting, crushing and processing of sugarcane result in a range of byproducts and sources of nutrient runoff and effluent wastewater being discharged into the Ba River. Wastewater from sugar mills have complex characteristics varying from mill to mill and often present a challenge due to a range of environmental concerns (Sahu & Chaudhari, 2015; Samuel & Muthukkaruppan, 2011). The processing of sugar in Fiji includes seven stages: collection of raw material, crushing, juice clarification, crystallisation, centrifuge, depolarization and conditioning and packing and bagging (Sahu, 2018). Through the crushing stage, sugarcane is washed with hot water to remove impurities. The impurities that accumulate between cutting and crushing include mud, oil and grease (Sahu, 2018). Sugar also consists of carbohydrates, protein, calcium, iron, potassium and sodium as well as a small percentage of heavy metals: arsenic, mercury, lead, cadmium, copper and zinc (Sahu, 2018). Through the juice clarification stage, the temperature is raised to 110–120 °C to catalyse the concentration of the sugar solution (Sahu, 2018). In the presence of moisture, sugar decomposes at 100 °C, cameralising and releasing water. On further heating, sugar changes to CO_2_ and H_2_CO_2_ (formic acid) which is a high acidity carboxylic acid (Sahu, 2018; Heitala, 2016). The Rarawai mill boiler also cycles water to cool the system. These processes involve hot water, heavy metals, formic acid and sugar crystals being discharged into the Ba River (Sahu & Chaudhari, 2015). This discharge of effluent has the potential to alter the pH affecting the rate of biological reaction and survival of various microorganisms within the Ba River and the connected downstream coastal reef ecosystems.

### Water quality sampling

**Fig. 2:**
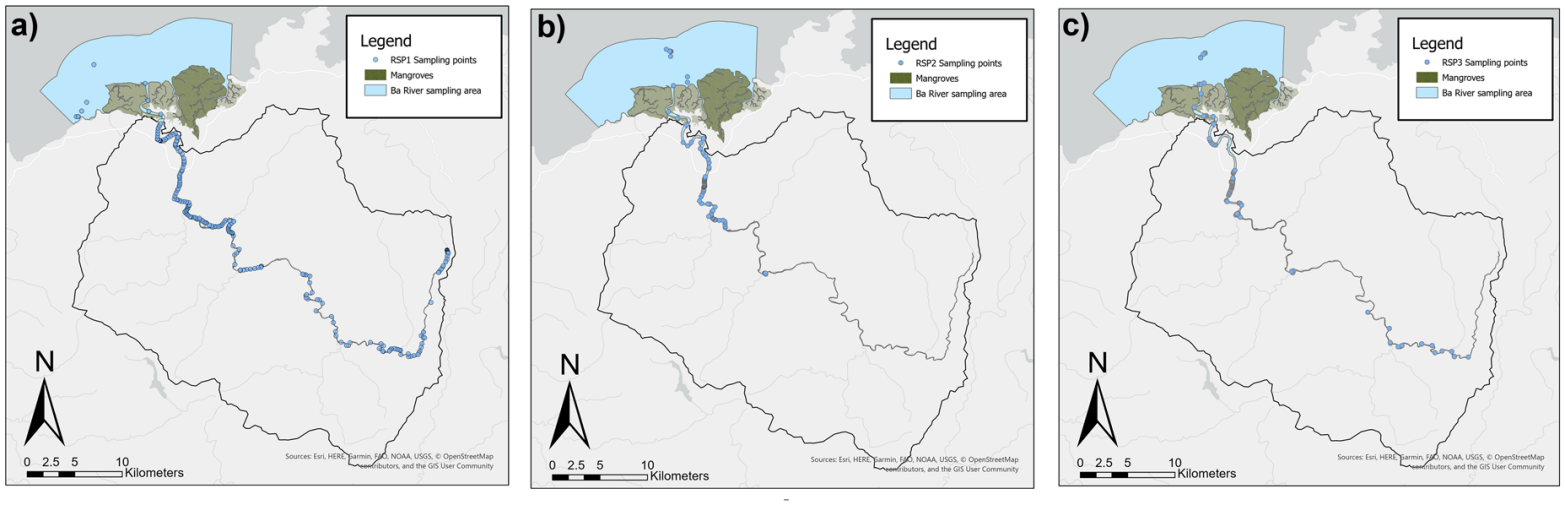
The maps show the Ba River catchment boundary on the island of Viti Levu, Fiji including the points of sampling during RSP1, RSP2 and RSP3, respectively. **a** RSP1 sampled 228 locations between 24 May and 4 June. **b** RSP2 sampled 117 locations on 22 June. **c** RSP3 sampled 311 locations between 12 and 14 October

As noted previously, the focus of this study was to characterise the spatio-temporal variation in pH along the Ba River using surface water quality measurements to identify potential trends and processes influencing acid and alkaline inputs. Analysis of pH data and identification of anomalous hotspots used a three point moving mean (Zimmerman & Kazandjian, 2003). Within this paper, hotspots have been defined as spatial zones corresponding to the greatest decline in pH per km distance along the surface water longitudinal gradient of Ba River and the coastal ocean. The identification of these hotspots may be used to inform more in-depth and longer-term water quality monitoring. The collection of water quality data was undertaken through a series of three rapid sampling periods (RSPs). These RSPs, detailed in Table 3, were intensive water quality measurement surveys along longitudinal gradients of the Ba River. The RSPs were undertaken before and during the sugarcane crushing season (RSP1, RSP2 and RSP3, respectively). The R2R approach required sampling water quality along a longitudinal gradient of 88.40 km with the upper boundary being the headwater springs of Ba River. The lower boundaries extended to the estuary and reef beyond the Ba River mouth. The RSPs collectively included 663 sampling points along The Ba River and its tributaries between April and December 2019 (Table 3). The sampling focused on the dry season and intensive agricultural harvesting dynamics without the flushing effects driven by heavy rainfall and flash floods common in the cyclonic wet season. RSP1 and RSP2 sampling included 352 water quality samples collected between May 24 and June 22. The sampling ranged from sea level to 584 m elevation. RSP3 included 311 water quality samples collected between 6 to 13 October. The Rarawai mill in Ba commenced sugarcane crushing on 9 July 2019 (FSC, 2020a, 2020b). Crushing ceased on 3 December 2019 before the arrival of Tropical Cycle Sarai on 28–29 December (FSC, 2020a, 2020b). The sampling period was limited by Tropical Cyclone Sarai (December 2019) and Tropical Cyclone Tino (January 2020). SP1 was used as a reference point from which all other sample points were measured along the 88.40 km length of the Ba River to the reef. At each of these sampling points, multiple water quality measurements were made using an Aquaread AP-2000 multiparameter probe. Each data point was geotagged for longitude (x), latitude (y) and altitude (z) as well as the time and date of measurement for RSP1, RSP2 and RSP3 (see Fig. 2a, b and c, respectively). The probe measured 14 parameters including temperature (°C), barometric pressure (mb), dissolved oxygen saturation (DO % saturation), dissolved oxygen concentration (DO mg/L), total dissolved solids (mg/L), turbidity (NTU), depth of measurement (m), pH, oxidation reduction potential (ORP), electrical conductivity (uS/cm @25 °C), resistivity (Ω Ohms.cm), salinity (practical salinity units), seawater specific gravity (σ_**T**_) and nitrate (mg/L).

**Table 3:** Rapid sampling period (RSP) campaigns

RSP	Timeframe	Data points	Spatial extent
RSP1	24 May–4 June 2019	228 sampling points	0.00–88.40 km from SP1
RSP2	22 June 2019	124 sampling points	55.20–88.40 km from SP1
RSP3	6–13 October 2019	311 sampling points	13.00–88.40 km from SP1

The surface water sampling approach followed the Standard Methods for Examination of Water and Wastewater (Greenberg et al., 1995; APHA, 1998; Chapman, 1996). pH was measured at surface-water median depths of 0.12 m, where possible, water quality was sampled from parts of the channel that were in well-mixed straight reaches. For the surveyed reaches, samples were retrieved at consistent intervals with a mean distance of 35 m depending on the riverine streambed composition and fluvial geomorphology at each sampling site. For the shallow reaches of the upper-catchment from the headwaters along river segments 1–6 (0 to 44.00 km), a grab sampling method was applied. This was the only sampling method possible for the upper-catchment due to the steep and often impassable streambank terrain. For the remaining downstream sites 7 to 13 (44.00–88.40 km) where the slope declined into the floodplain and estuary, water quality measurement was conducted using a raft with the same multi-parameter probe for recording water quality data points. Noting the different sampling designs of each of these studies, this comparison cannot be generalised.

Within the floodplain and the marine zones, both RSP1 and RSP2 identified anomalies in pH levels with trends in pH values declines across 3 longitudinal points. Slope of the decline is defined as *β*_1_in Eq. (1):

1 $$y = \beta_{{\mathbf{0}}} + \beta_{{\mathbf{1}}} x,$$

where *β*_0_ = *y* intercept, *β*_1_ = slope and *x* is the pH value.

The rapid sampling strategy was designed to measure pH across both space and time. Each discrete sampling period, or RSP, focused on measuring the longitudinal spatial variability in water quality. The comparison amongst RSPs yielded insights into temporal variability in water quality. Water quality, including pH, is influenced by a range of temporal variables: time of sampling, total duration of sampling and tidal influences on tidal mixing fluxes (diffusion and advection), tidal influences, rainfall and runoff as well as fluctuating input volumes of point-source contamination within waterways. To minimise variability, sampling was undertaken between 06:00 and 18:00 each day to maintain mean surface water temperature as close as possible to 25 °C. Temperatures of sampled surface water ranged from 18.75 to 29.90 °C with a mean of 26.67 °C and a standard deviation (SD) of ± 1.79. To minimize tidal variations, each RSP measurement occurred during the same point of the ebb tide, the only opportunity to navigate the shallow channel. A hydrograph was set up in the Ba River to verify the extent of tidal influence. High resolution temporal variability of pH in the Ba River was measured every 2 min using data-loggers at an upstream site located in the central floodplain within Ba River segment 9 (57.60 km downstream of SP1) and at a downstream site at a Ministry of Waterways monitoring station located between the coastal floodplain and estuary within segment 11 (70.20 km). During the before the sugarcane crushing season (RSP1), samples were collected daily over a two week period which had a cumulative recorded rainfall of 1.50-mm (Fiji Meteorology Services, pers comm, 2019).

### Geospatial analysis

Geospatial Information Systems (GIS) and remotely sensed satellite imagery have been widely applied within environmental monitoring and assessment (Dewan & Yamaguchi, 2009; Larsen, 1999; Machiwal et al., 2011; Zhang et al., 2008). Within this study, geospatial analysis served three functions: (1) to overlay multiple layers of ancillary data over the Ba Catchment to delineate the 13 segment framework that has been applied here, (2) to visualise the results of water quality monitoring for RSP1, RSP2 and RSP3. All geospatial layers were projected within the Universal Transverse Mercator (UTM) and World Geodetic System (WGS) 94 coordinate systems; and (3) geostatistical interpolation of the results to visualise a model of pH values across the entire surface water body within the study area. The water quality data points were interpolated using the kriging geostatistical method. The kriging method is based on statistical models and autocorrelation of the relationships amongst measured points to generate a prediction surface (Vieux, 2016). The general formula for the interpolation is formed as a weighted sum of the data:

2 $$Z(S_{0} ) = \mathop \sum \limits_{i = 1}^{N} \lambda_{i} Z(S_{i} )$$

where, *Z*(*S*_i_) = the measured value at the ith location; *λ*_i_ = an unknown weight for the measured value at the ith location; *S*_0_ = the prediction location; *N* = the number of measured values.

The river buffer and coast buffer were merged to form the surface extent for the kriging interpolation. The standardized river outline vector shapefiles were downloaded from the official dataset of the Ministry of Lands and Mineral Resources of Fiji (Ministry of Lands, 2019). The river shapefiles were extended from 70 km to cover the total 88.40 km covered during the RSPs. A 500 m buffer was generated along the length of the river made up of 250 m on the left banks of the Ba River. A coastline buffer of approximately 90 km^2^ was generated extending 8.5 km from the river mouth along the coastline to the east and west and 5 km seaward. Slope and elevation were modelled through a Digital Elevation Model (DEM) generated using publicly available data from Shuttle Radar Topography Mission (SRTM) databases. The results of the DEM highlight the point where the floodplains meet the mountains. The DEM-derived topographical data were used to generate the boundaries of the Ba Catchment, the upper-catchment, lower-catchment floodplain, the marine zone and the 13 segments described in Tables 1 and 2 (EPA, 2019). The Strahler stream ordering algorithm was used to define and categorise perennial and ephemeral tributaries flowing into the river. Geo-referenced geology maps were overlaid onto the satellite image of the Ba Catchment (Rodda, 1966). The Ba estuary mangrove shapefiles were digitized using Sentinel 2 and Landsat 8 datasets through the Earth Engine data catalogue.

Visualising water quality data involved transferring sampling points gathered in the field to a GIS grid. The AP-2000 water quality probe geotagged each sampling point with x, y and z dimensions with ± 10 m accuracy. The data were processed by converting the location of each sampling point location to the UTM WGS84 coordinate reference system and then uploaded to an ArcGIS database. The datasets for RSP1, 2 and 3. The RSP1, 2 and 3 data were collated in RStudio and Excel and analysed using one-factor ANOVA and *t* tests.

## Results and discussion

### Spatial variability in pH

**Fig. 3:**
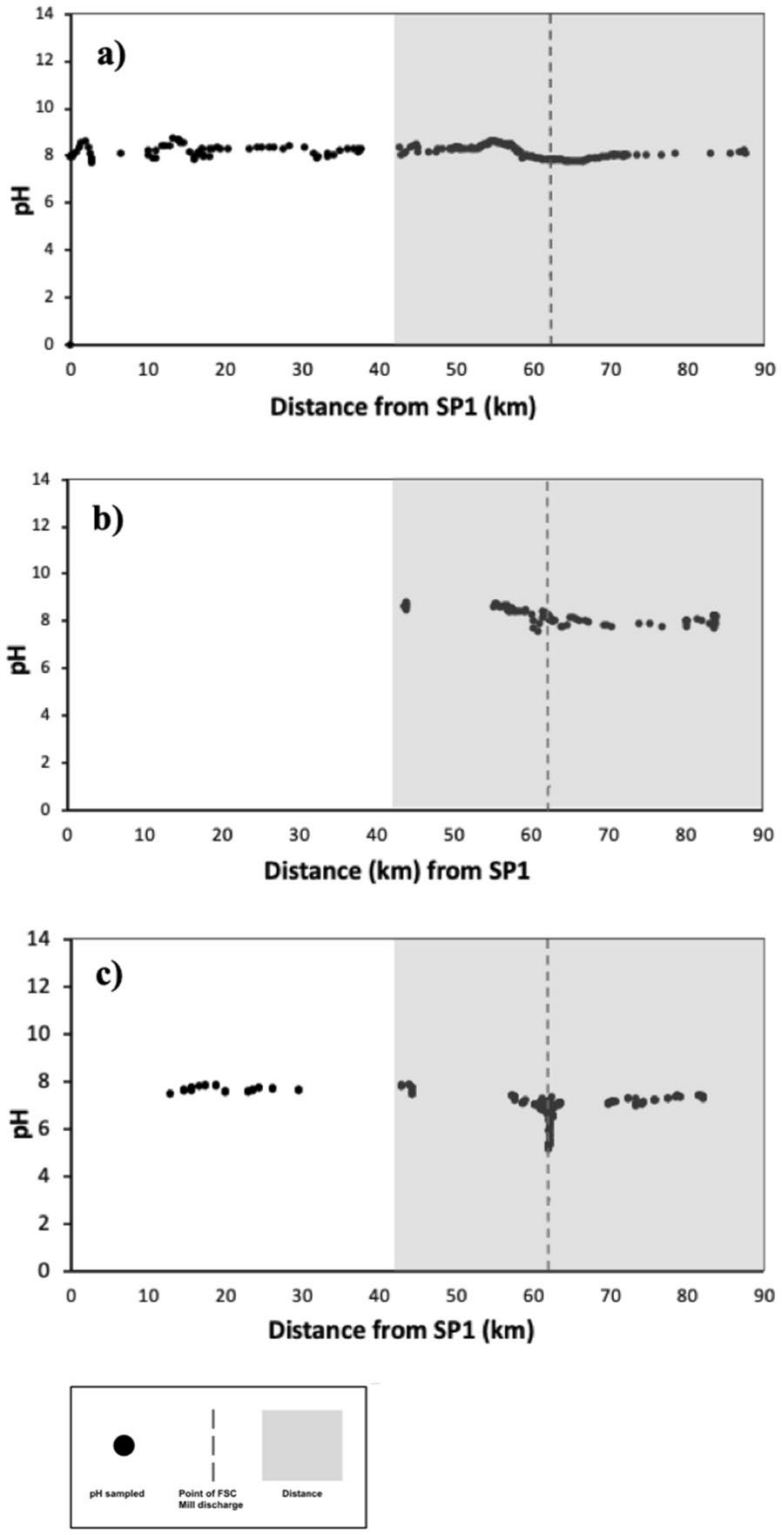
pH observations made at intervals along the Ba River starting from ridge sampling point 1 (SP1) (S17.609320° E177.933518°) extending to reef margins (at approximately S17.417052°, E177.595095°). The dashed line at 62.19 km downstream of SP1 indicates the location of the FSC Mill discharge. **a** RSP1 was conducted between 24 May and 4 June 2019, before the sugarcane crushing season. **b** RSP2 was conducted on 22 June 2019, later in the agricultural cutting stages but still before the sugar cane crushing season. **c** RSP3 was conducted from 6 to 13 October 2019 during the crushing season, the only RSP survey with point source discharge from the FSC Mill. The shaded subsample distance between 44.00 and 90.00 km from SP1 is displayed in higher resolution in Fig. 4

In general, the results document spatial variation trends along the Ba River, and temporal variability amongst the three rapid sampling periods before (RSP1 and RSP2) and during (RSP3) the sugarcane crushing season. Observed pH varied along the 88.40 length of the Ba River as mapped in Fig. 2 and plotted in Fig. 3. For RSP1, the mean pH of the upper catchment (0 to 44 km) had a mean pH of 8.19 compared to a mean pH of 8.14 in the lower catchment between 44.00 and 90 km. While sampling in the upper-catchment within this study used a grab-sampling technique which may have resulted in greater variation than the research raft monitoring methods used in the lower-catchment, the results remained consistent with the findings of other studies of Ba Catchment. Both Qamese (pers comm, 2018) and Fagan et al. (1995) found pH values ranging from 7.20 to 9.10 in the upper Ba Catchment. The pH values in the lower catchment were found to have a lower mean pH range: 7.60 (Tamata and Kubuabola., 1993) and 7.40 (Qamese pers comm, 2018). In this study, the greatest decline in pH values equalled 3.06 pH units between the coastal floodplain and the ocean (segments 7 to 13, Fig. 3 and Table 4).

**Table 4:** pH trends for RSP1, RSP2 and RSP3 surveys. For RSP3, the pH declined by 3.06 pH units between segments 7 and 11, a decline at a rate of − 0.17 pH units per km. By contrast, the maximum decline measured in RSP1 and RSP2 were 0.78 and 1.17 pH units, respectively, equivalent to a − 0.035 and − 0.070 pH units decline per km, respectively

pH hotspots	RSP1, 228 obs*	RSP2 124 obs*	RSP3 311 obs*
Mean upstream pH	8.51	8.75	8.38
Minimum pH	7.73 @ 66.47 km	7.58 @ 60.85 km	5.32 @ 62.15 km
Mean downstream pH	8.22	8.21	7.34
Overall mean	8.16 (0.27)	8.20 (0.25)	6.94 (0.53)
Standard deviation	0.27	0.25	0.53
Amplitude	0.78	1.17	3.07
β_1_ upstream (pH unit • km^−1^)	− 0.035	− 0.070	− 0.172
β_1_ downstream (pH unit • km^−1^)	0.023	0.028	0.101

### pH variability measured before the crushing season (RSP1 and 2)

Before the crushing season, for RSP1, the pH value of 8.51 declined by − 0.04 pH units per km from the foothills to the beginning of the floodplain in segment 7 (starting at 44.44 km) to a minimum 7.75 pH at 3.17 km downstream of Ba Town Bridge. Continuing downstream from this minimum, pH increased by 0.02 to reach 8.22 at 87.50 km downstream, adjacent to the reef ecosystems of Ba. The RSP2 survey results were consistent with the RSP1 results. Over the same length of Ba River, the mean pH range declined from 8.75 (44.12 km) by − 0.076.99 × 10^–2^ pH units per km to 7.58 within 0.65 km upstream of the old Ba Town bridge at 60.85 km. The pH rose again by 0.03 pH units per km towards 8.21 pH close to the reef ecosystems at 83.71 km. Both of these rapid sampling periods identified declines in pH between the lower agricultural floodplain and the estuary: segments 7 and 11 between 44.00 and 73.00 km downstream of SP1. The spatial extent of pH declines identified through RSP1 and RSP2 occurred between segments 10 and 11 (61.70 to 73.00 km) near Ba Town and the sugar mill.

Why might pH values decline throughout these particular reaches of Ba River? A number of potential factors influence the equilibrium balance of acid and alkaline inputs. From the headwaters to the beginning of the floodplain 44.00 km, the primary lithologies comprise the Ba Volcanic Groups including basaltic and derived flows: Pliocene undifferentiated andesitic flows and pyroclastics, pockets of intrusive andesitic rocks and Pleistocene basalt-derived sandstone with limestone (Kumar, 2005; McPhie, 1994; Rodda, 1966). Past the 44.00 km point, the dominant lithologies along the riverbanks are composed of Pleistocene alluvial surficial deposits as sands, gravel and silts (Rodda, 1966). The basalt and alkaline limestone carbonate inputs correlated with higher pH ranges in the upper Ba Catchment (Norton et al., 2001). Similarly, pH ranges were also found to rise towards the open ocean and the carbonate reef. Riparian vegetation has a buffering capacity linked to reducing acidifying inputs into waterways (Peterjohn & Correll, 1984). In terms of vegetation, the mangrove forest composed of *R. stylosa*, *R. mangle* and *R. selala* (Ellison & Fiu, 2010) extends along Ba River from 72.50 km to the ocean at 79.20 km. The shade, decreased temperatures and photosynthesis from riparian vegetation are associated with higher levels of dissolved oxygen correlated with increased pH (Boto & Bunt, 1981). Mangrove vegetation has been linked to decreased acid-soluble metal concentrations (Zhou et al., 2010). Tidal influences have also been linked to pH fluctuations (Ovalle et al., 1990). In the Ba River Catchment, the tidal zone is complex and could be defined by water chemistry, salinity, vegetation, topography and the spatial range of tidal influence on fluctuating river depths. During the RSP1 survey, electrical conductivity (µS/cm at 25 °C) and salinity (PSU) increased by an order of magnitude from freshwater zones to brackish downstream of Ba Town bridge at 62.70 km (1.00 × 10^3^ µS/cm and 0.5–1 PSU) and then increased by another order of magnitude (1.00 × 10^4^ µS/cm and 1–10 PSU) at 71.50 km, at the edges of the riparian and estuarine delta mangroves. However, tidal influence of flows reportedly reached as far as the agriculturally intensive areas of the central floodplain, at 55.00 km. Topography and bathymetry also affect the intertidal zone range. At the foothills, 42.42 km, the elevation decreased from a mean range of 40 m to 15 m elevation. At 59.20 km, just upstream of Ba Town, the elevation dropped from 10 m to sea level extending along the remaining 23.20 km along the river gradient towards the river mouth. Studies have also linked the effects of point and diffuse source pollution on waterways, influencing water quality and the presence of bio-indicators such as sensitive benthic fauna (Stevenson et al., 2012; Neumann & Dudgeon, 2002; Lenat, 1984). Agricultural runoff commonly increases nutrient inputs (including N and P) linked to algal blooms and eutrophication processes which reduce pH (Cooke & Prepas, 1998; Hoorman, et al., 2008). During the RSP1 and RSP2 surveys, the minimum pH values were consistently measured near Ba Town. The minimum pH of RSP1 (7.75) was within 0.8 km of the Ba Town bridge and the minimum pH measured in RSP2 was within 0.65 km upstream of the old Ba Town bridge. Diffuse source pollution includes runoff from the urban and paved areas of segment 10 (61.70 to 63.30 km) as well as intensive agricultural areas (sugarcane) extending from segments 8–11 (50.00 to 73.00 km). There are some smaller plots of agricultural land adjacent to Ba River in segments 6 and 7 (31.80–50.00 km).

### Identifying pH hotspots during the crushing season (RSP3)

**Fig. 4:**
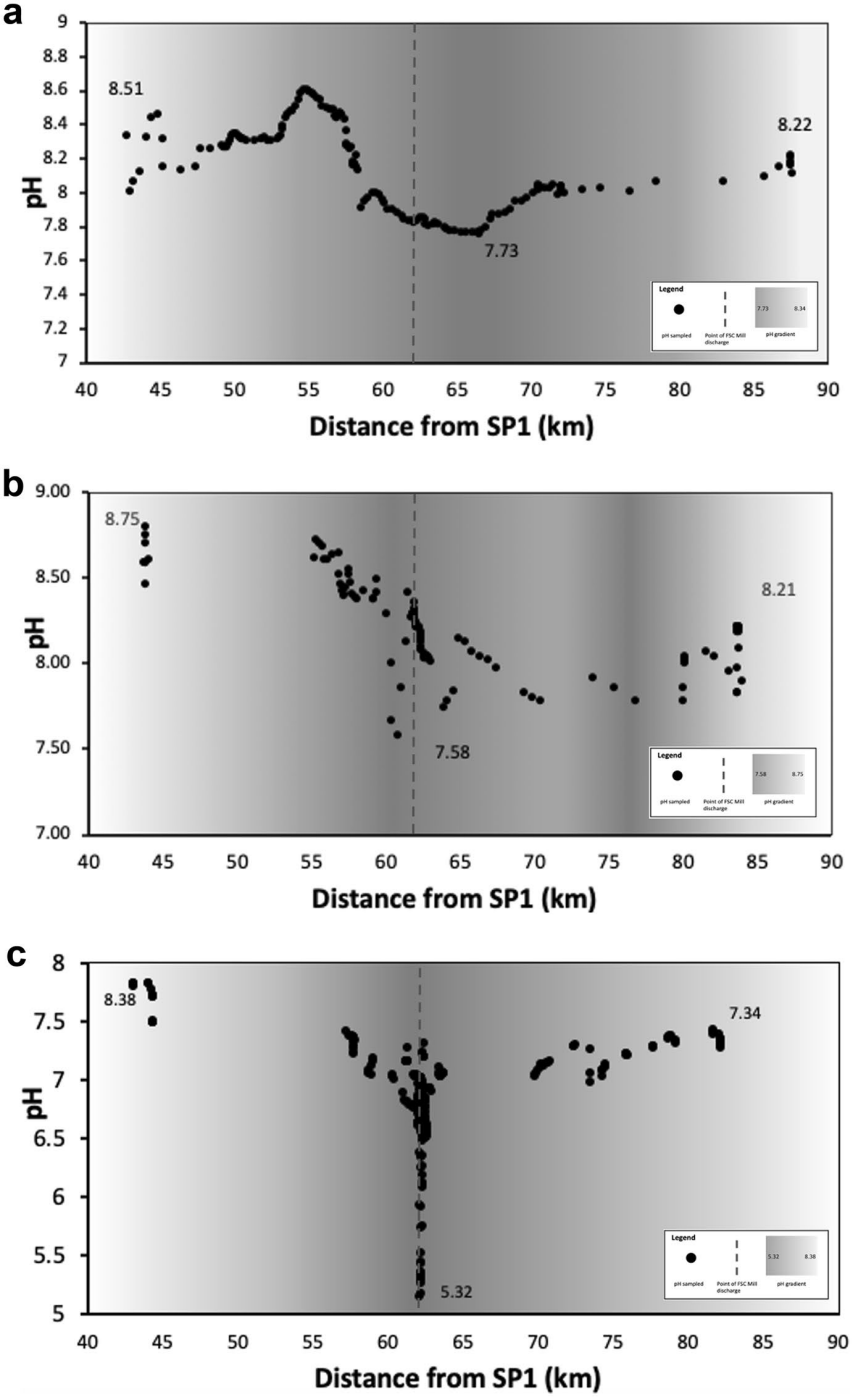
Subsampled distance of pH observations made at intervals along the Ba River in Fiji at distances extending from 44.00 to 90.00 km downstream of SP1. The point of FSC Mill discharge is outlined with the dotted line at 62.19 km. The pH gradient is highlighted using a colour gradient with lower pH visualised with darker colours and higher pH highlighted in white. **a** For RSP1, mean pH was 8.15 (SD ± 0.27) with a range of 7.73 to 8.51 pH. The pH minimum was identified at 66.47 km with a minimum pH of 7.73. **b** For RSP2, mean pH was 8.20 (SD ± 0.25) with a range of 7.58 to 8.75. The pH minimum was identified at 60.85 km with a minimum pH of 7.58. **c** For RSP3, mean pH was 6.94 (SD ± 0.53) with a range of 5.32 and 8.38. A pH hotspot was identified at 62.15 km near the FSC mill with a minimum pH of 5.32

Based on the pH minimum associated with Ba town identified in the RSP1 and RSP2, the area highlighted in grey in Fig. 3 (from segments 7 to 13) was targeted for further sampling in the RSP3 survey during the sugar cane crushing season as shown in Fig. 4. The minimum values in pH for RSP1 and RSP2 were identified within 1 to 5 km from the point-source effluent discharge from the FSC mill. To explore the sensitivity of water quality to this point-source discharge from the FSC mill, the RSP3 survey took a total of 311 river samples along the 69.13 km stretch extending from 13 km below SP1 to the reef. Out of the total 311 samples (segments 2 to 13), 260 were focused on monitoring pH areas within segments 9 to 13 (57.60 to 88.40 km). Within segments 9–11, the sampling was undertaken at a higher spatio-temporal resolution (see Fig. 4c). Temperatures of sampled surface water ranged from 23.60 to 30.75 °C with a mean of 28.18 °C (SD ± 1.11).

During this sugarcane crushing season (RSP3), the mill abstracted water from Ba River and pumped wastewater directly back into the river at km 62.19. The sampled length of RSP3 was 13.00 km from the origin. The lower reef boundary was sampled at a maximum 82.13 km. The decline in pH identified close to the point-source discharge from the FSC sugar mill intensified between RSP1 and RSP2 conducted before the sugarcane crushing season and the RSP3 survey conducted during the sugarcane crushing season. The mean pH along the Ba River ranged between 7.58 and 8.80 before the sugarcane crushing season for RSP1 and RSP2, respectively. The overall mean pH from RSP1, 8.16 (SD ± 0.23), did not differ significantly from the mean pH of RSP2, 8.20 (SD ± 0.25) with an overall mean of 8.18 (SD ± 0.30). For RSP3, pH values ranged from 5.32 to 8.38. The mean pH was 6.94 (SD ± 0.53). Within the subsampling region of interest (floodplain to ocean) from segments 7 to 13 (distances 44.00 to 88.40 km), pH declined by − 1.71 ×  − 10^–1^ pH units per km from 8.38 at 44.30 km to the point of minimum pH (5.32) at 62.15 km. The minimum pH values were recorded within 40 m of the point of FSC mill discharge. From this minimum, pH increased by 1.01 ×  − 10^–1^ pH units per km longitudinally to 7.34 at 88.40 km.

The sugarcane crushing season and point-source discharge from the sugar mill resulted in a dramatic 3 pH unit decline between 56.6 and 73.0 km corresponding to segments 9 to 11. ANOVA analysis of pH values for RSP1, RSP2 and RSP3 resulted in an *F* value of 771.57 with a *P* < 0.001. ANOVA pairwise analyses of pH values for RSP1, RSP2 and RSP3 results reveal statistically significant differences between RSP3 pH values and both RSP1 and RSP2 pH values. The mean pH value for the RSP3 crushing season survey was 6.94 compared to the combined mean pH value of 8.18 for the RSP1 and RSP2 surveys. By every statistical analysis conducted, including *t* test and ANOVA, the RSP3 crushing season survey mean pH value was significantly lower than the mean pH values of both the RSP1 and RSP2 before crushing season surveys (*P* < 0.001).

Other environmental impact assessments of water quality in the Ba River have found a range of point-source and diffuse source inputs linked to water quality changes. The assessment conducted by Fagan et al. (1995) before and during the crushing season highlights the potential influence of the Ba FSC mill discharge as a key source of pollution in Ba River. However, the environmental impact assessment results noted that the FSC mill was not the only major source of disturbance. Runoff from Ba Town and wider agricultural runoff were also identified as potential sources influencing water quality in the lower reaches of the Ba River. The assessment of Tamata and Kubuabola (1993) before the crushing season identified high concentrations of ammonia and nitrite likely resulting from agricultural runoff. Sewage discharge and faecal coliforms near Ba Town are also identified by various assessments (Anderson et al., 1999). Through the rapid sampling periods’ experimental design, this study builds on these past environmental assessments in identifying the influence of smaller scale pulsing spatio-temporal pH declines within each of the 13 R2R segments. These declines are most clearly identified between segments 9–11 (56.60–73.00 km) as observed in RSP3.

The study provided the opportunity to compare different methodologies: sampling with fixed sensors over time and spatial sampling with and without a raft. Some variation can be attributed to sampling methods: RSP1 in the upper-catchment (0–44 km downstream) without a raft resulted in a standard deviation of 0.30 compared to the lower catchment standard deviation of 0.25 in the floodplain and marine zones (44–90 km). These results highlight the potential influence of different water quality sampling methods in the upper and lower catchment. However, in the lower-catchment, where water quality was always sampled from the raft, the temporal variation increased with discharge from the FSC mill. The mean lower-catchment standard deviation of RSP1 and RSP2 was 0.25 compared with 0.53 for RSP3, with FSC mill discharge.

### How does the crushing season pH hotspots compare to seasonal variability in pH?

It is important to explore how these spatial trends before (RSP1 and 2) and during (RSP3) the sugarcane crushing season fit within wider seasonal and spatio-temporal river catchment processes of hydrology and phenology (Comber & Wulder, 2019). Considering seasonal context reduces the risk of conflating observed pH trends with patterns that could have arisen from natural fluctuations or from sampling errors (Oaten, 1996). In order to understand these spatial phenomena within the wider temporal diurnal and seasonal catchment processes, the observed spatial decline in pH associated with the RSP3 crushing season survey was assessed against temporal trends. The two stationary data sensors logging the longer-term high frequency time-series were located at 57.7 km and 70.2 km downstream of SP1. These sensors shed light on trends upstream and downstream of the Rarawai sugar mill, located at km 62.19 km downstream of SP1. The stationary data loggers documented higher resolution temporal variability between the RSPs and provide more detailed insights into the seasonal variability of pH. These data loggers recorded pH and temperature at 2-min intervals. At the upstream site located in the central floodplain within segment 9 at km 57.60, the mean diurnal pH amplitude was 0.31. At a downstream site, the Ministry of Waterways monitoring station located between the coastal floodplain and estuary within segment 11 at km 70.20, the mean diurnal pH amplitude was 0.46. The pH measurements made downstream of the sugar mill were significantly lower than pH measurements made upstream of the sugar mill (ANOVA, *P* < 0.001). ANOVA analysis of the daily mean pH value for the upstream and downstream sites, over 73 days, resulted in an *F* value of 6.86 and a *P*  ≥ 0.001 indicating that the difference between upstream and downstream sites was not significant in comparison to the spatial variability closer to the identified hotspot point.

**Fig. 5:**
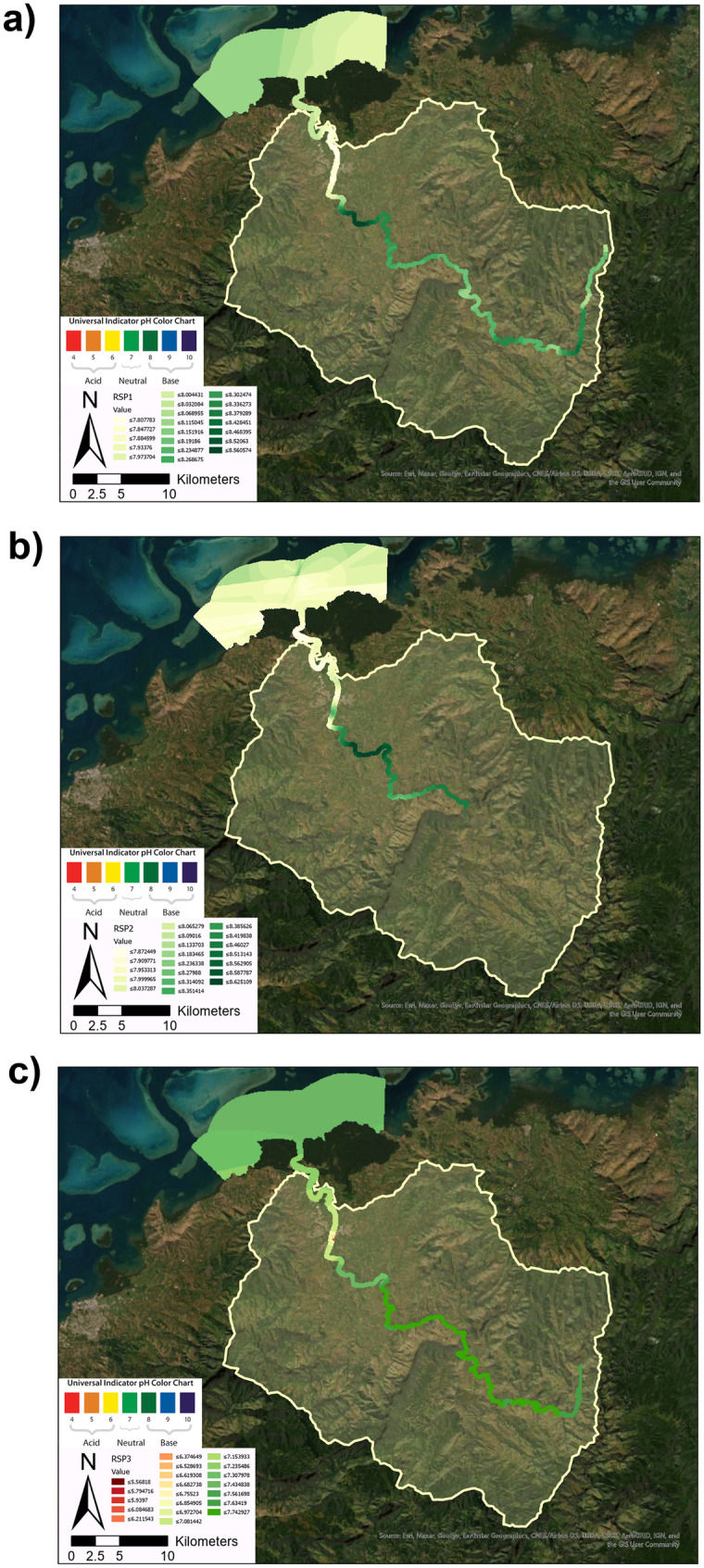
The pH values shown in Fig. 3 are visualised for the Ba River using Kriging geostatistical analyses. **a** displays the pH values for RSP1. **b** displays the pH values for RSP2. **c** displays the pH values for RSP3

The pH decline across both monitoring stations indicate that mean diurnal pH amplitude was 0.39 and the mean seasonal pH amplitude was 2.01. The term amplitude captures the full variability between the maximum and minimum value calculated as a three-point mean. The pH declines downstream of the mill were 0.78, 1.17 and 3.06 for RSP1, RSP2 and RSP3, respectively. The pH declines were 2, 3 and 7.85 times greater than the mean diurnal amplitude for RSP1, RSP2 and RSP3, respectively. The pH declines were 0.39, 0.58 and 1.52 the proportion of mean seasonal amplitude for RSP1, RSP2 and RSP3, respectively. The pH decline of the RSP3 crushing season hotspot (Fig. 4c) far exceeded both diurnal and seasonal amplitude as visualised in Fig. 5.

**Fig. 6:**
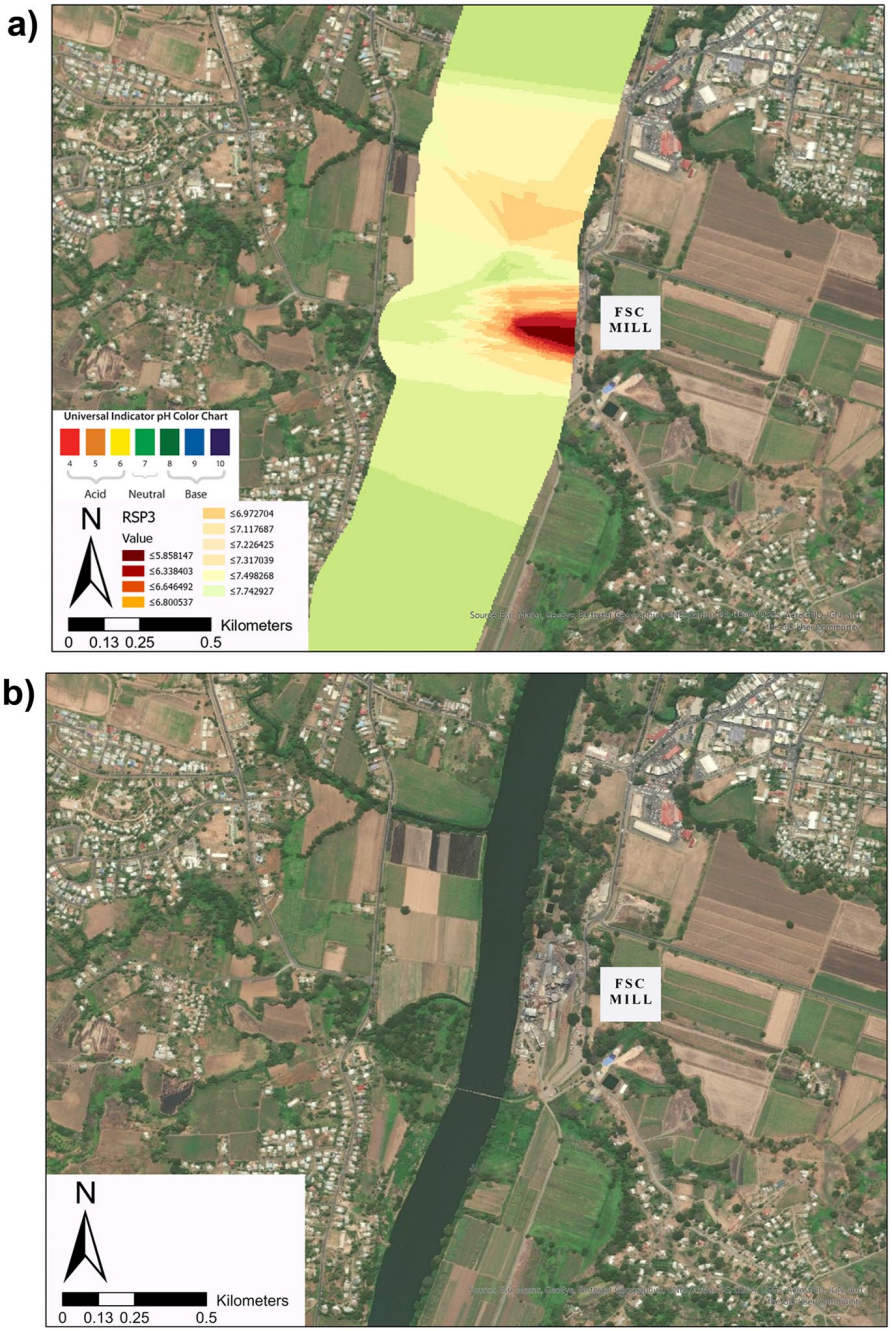
The location of the pH decline ‘hotspot’ identified within RSP3 is shown in **a**. The hotspot is located in segment 11 (between 61.00 and 63.00 km downstream of SP1). This site correlates with the FSC Sugar Mill as well as town runoff **b**

The identification of a pH hotspot of magnitudes exceeding diurnal and seasonal trends in pH within this study builds on the other studies and environmental impact assessments of Qamese (pers comm, 2018); Fagan et al. (1995) and Tamata and Kubuabola (1993) undertaken within the Ba Catchment. These findings are also comparable to other catchments throughout Fiji. Other studies have attributed fluxes in riverine surface water quality to a range of environmental health and pollution factors. As shown in Fig. 6, the hotspot is near the FSC mill located at km 62.19 close to previously identified point source pollution from the FSC mill in Ba (GEF, 2007; Falkland, 2002). Similar reports of FSC mill discharge have been observed from the FSC mill of Labasa into the adjacent Qawa River on the island of Vanua Levu (Janine, 2010; Tuivanualevu, 2017; Silaitoga, 2009). A study by Bainivalu (2015) observed over a limited period that the FSC mill in Labasa also altered the natural pH level of the receiving water body. Similar trends were observed in the Ba River, with a decline in pH identified adjacent to the mill. However, in the Ba River case, this decline occurred despite the expected buffering effect of the tidal zone extending further upstream in the Ba River to km 55.00. Alongside these interesting spatial results, the study also finds the temporal trend in which mean pH declined from the before (RSP1 and RSP2) to during the sugarcane crushing season (RSP3). This trend may show a correlation between the cumulative effects of an increased rate or volume of point-source effluent discharge and/or agricultural runoff and acidity overwhelming the buffering capacity. However, further studies are needed to test this correlation. Similar findings in the Ba and Labasa Catchment are to be expected given the shared characteristics of both being sugarcane-intensive catchments located in the northwestern rainshadow areas. Wider-scale studies using remote sensing and land cover classification observe that the sugarcane belt covering Ba River catchment and the adjacent Tavua, Lautoka and Nadi areas all experience increased nutrient and sediment loading in river basins (Dadhich et al., 2020). Within this study, increased nutrient and sediment loading observed through satellite imagery is largely attributed to sugarcane agriculture inputs. The variability in ranges of pH between the highland and lowland areas identified in this study of Ba Catchment has also been observed across other catchments in Fiji. For example, water quality in the upper reaches of Rewa river catchment has met stringent pH surface water quality criteria over several assessments (Naidu, 1988; Naidu & Brodie, 1987). However, more recently, high concentrations of nutrients (Carpenter and Lawedrau, 2002) and higher than average regional levels of accretion (Terry, et al., 2002; Terry Kostaschuk, 2001) were found in the lower reaches and estuary of Rewa. These changes were attributed to a range of land use change factors as well as cyclones, floods and associated fluvial-geomorphological change over time (Printemps, 2008; Ram, 2013). Many of these studies also cited the impacts of sugarcane agriculture (Bainivalu, 2015; Dadhich et al., 2020; Terry and Garimella, 2008). These factors are also highly characteristic of the Ba River Catchment.

Despite focusing on the Pacific SIDS R2R program, this study yields results comparable to other studies based on continental sampling. Such studies have found variable impacts of sugarcane agricultural runoff and sugar mill discharge. These sources of contaminants have been found to affect stream pH either making it too acidic or alkaline (Tucker & Robinson, 1990). Past studies have found sugar mill effluent to have a pH of 4.2 (Sajani & Muthukkaruppan, 2011), 4.55 and 5.25 (Baskaran et al., 2009), 4.5 (Matkar & Gangotri, 2002) and a range of 6.0–7.6 (Kumar et al., 2001). A study by Kolhe et al. (2008) observed a range of 6.5 and 7.0 of untreated effluent and that of treated effluent as 7.5 and 7.5 in November and December, respectively. Turinayo (2016) found that in Musamya River, Uganda, discharge from sugar mill effluent correlated with an instream pH of 5.6 compared to an upstream pH value of 7.1. Gunkel et al. (2007) found that in the Ipojuca River, Brasil, discharge from sugar mills correlated with a pH decrease from 6.7–6.2 linked to influences of fertigation. Other studies found positive uses for sugar mill effluent discharge including Khan (2019), in Pakistan, whose study linked sugar mill effluent discharge onto nearby soils to increased yield of certain crops. It is widely accepted within the literature that agriculture is one of the largest contributors to non-point source water pollution (Batie, 1983). Other studies have highlighted the impacts of industrial runoff and urban pollution, having similar influences on lowering pH (Morrison et al., 2001; Olajire and Imeokparia, 2000). These sources of pollution have also been identified as potential factors influencing the occurrence of pH declines found in this study and in other past studies of Ba Catchment (Fagan et al., 1995; Tamata & Kubuabola, 1993).

The study combined the GEF framework’s spatial focus on the R2R context with temporal dimensions including the impact of the sugar cane crushing season and the diurnal cycle of pH within the context of monitoring for SDG target 14.1 and 15.1. Through this monitoring, we have highlighted a potential scientific pathway to better connect environmental monitoring of water quality pollutant hotspots across land-sea boundaries for Pacific SIDs contexts.

## Conclusions

In the Ba River catchment on Fiji’s largest island Viti Levu, pH declined by an estimated 0.80 pH units drop between the upper and lower catchments during the sugarcane growing season. The decline in pH was amplified by an order of magnitude to 3.06 pH units during the RSP3 sugarcane crushing season. The greatest decline in pH occurred within 40 m of the point-source discharge of the FSC mill during the sugarcane crushing season.

The results of this study add to the emerging literature on water quality sampling in R2R contexts in Pacific SIDS. Currently, this literature is still relatively limited to grey literature, baseline environmental assessments and project reports. By observing the spatial variability of pH along the length of Ba River, this study has shed light on a decline in pH defined within this study as a ‘hotspot’ area where longer-term seasonal monitoring is required. The study demonstrated the success of a focused rapid sampling. Through three rapid sampling periods, river catchment longitudinal variability identified a spatio-temporal pH hotspot along the Ba River. The results of this comparison show significant spatial and temporal variability in pH with the hotspot associated with the FSC mill point source discharge, Ba town runoff and wider agricultural runoff. These trends must be understood within wider spatio-temporal river catchment hydrological processes. The statistically significant decline in pH induced by the FSC Sugar mill was 2, 3 and 7.85 times greater than the diurnal amplitude for RSP1, RSP2 and RSP3, respectively. The pH hotspot decline was equivalent to 39%, 58% and 152% times the mean seasonal amplitude for RSP1, RSP2 and RSP3, respectively. The decline in pH adjacent to Ba Town and the FSC mill exemplifies critical spatiotemporal variability, highlighting the need for longer-term temporal monitoring.

The experimental methods in this study highlight how rapid water quality sampling across long distances within short-time frames can be used to highlight spatial variability and earmark hotspots that can then be addressed with further targeted longer-term temporal water quality monitoring. The study also highlights a potential low-cost and time-effective investment to optimise insights within the broader context of limited data availability. In doing so, the study provides an example for rapid water quality sampling that contributes to holistic spatio-temporal systems-based environmental monitoring. This approach would provide greater granularity to enhance our understanding of the state of coastal systems and assessments in the Pacific R2R context.

As set out in the objective of this study, our study serves as foundational groundwork to identify spatial and temporal hotspot areas requiring further focused long-term monitoring. This study also adds to the growing body of research on the interplay between the intensification of agriculture and the environment in Pacific SIDS.

## Data Availability

The Ba watershed data collected in this study is archived at the British Oceanographic Data Centre and is available at the following link: https://www.bodc.ac.uk/data/published_data_library/catalogue/10.5285/cd857050-0c6d-3a71-e053-6c86abc08527/  This data can be cited as Metherall N.; Holland E.A.; Beavis S.; Vinaka A.M.D. (2021). Chemical water quality in the Ba Catchment coastal zone - 2019 - University of the South Pacific. NERC EDS British Oceanographic Data Centre NOC. doi.org/10/gzcg.

## References

[CR1] Abdussamatov H, Khankov I, S., & V. Lapovok, Y. (2012). The thermal inertia characteristics of the system ocean-atmosphere. Journal of Geographic Information System.

[CR2] Anderson, E., Cakuase, N., & Fagan, L. L. (1999). Effects of multiple resource use on water quality in the Ba river and estuary. Suva.

[CR3] ANZECC & ARMCANZ. (2000). Australian and New Zealand guidelines for fresh and marine water quality The Guidelines Australian and New Zealand Environment and Conservation Council Agriculture and Resource Management Council of Australia and New Zealand. *National Water Quality Management Strategy*, *1*(4), 314. Retrieved from http://www.dofa.gov.au/infoaccess/

[CR4] APHA-AWWA-WEF. (1998). No Title (20th ed.). Retrieved from https://www.worldcat.org/title/standard-methods-for-the-examination-of-water-and-wastewater/oclc/779509419

[CR5] Asian Development Bank. (2014). Regional state of the Coral Triangle - Coral Triangle marine resources: Their status, economies, and Management. Retrieved from http://www.adb.org/sites/default/files/publication/42393/regional-state-coral-triangle.pdf

[CR6] Aufdenkampe AK, Mayorga E, Raymond PA, Melack JM, Doney SC, Alin SR, Yoo K (2011). Riverine coupling of biogeochemical cycles between land, oceans, and atmosphere. Frontiers in Ecology and the Environment.

[CR7] Bainbridge Z, Lewis S, Bartley R, Fabricius K, Collier C, Waterhouse J, Brodie J (2018). Fine sediment and particulate organic matter: A review and case study on ridge-to-reef transport, transformations, fates, and impacts on marine ecosystems. Marine Pollution Bulletin.

[CR8] Bainivalu, A. (2015). The microbial profiling of the Qawa River using molecular methods by (University of the South Pacific). Retrieved from http://digilib.library.usp.ac.fj/gsdl/collect/usplibr1/index/assoc/HASH0191/18d7f9ab.dir/doc.pdf

[CR9] Baker-Médard M, Allnutt TF, Baskett ML, Watson RA, Lagabrielle E, Kremen C (2019). Rethinking spatial costs and benefits of fisheries in marine conservation. Ocean and Coastal Management.

[CR10] Baskaran, L., Sundaramoorthy, P., Chidambaram, A. L. A., & Ganesh, K. S. (2009). Growth and physiological activity of greengram (Vigna radiata L.) under effluent stress. *Botany Research International*, *2*(2), 107–114. https://www.academia.edu/download/55035994/10.pdf

[CR11] Batie, S. (1983). Resource policy in the future: glimpses of the 1985 Farm Bill. Farm and Food Policy Critical Issues for Southern Agriculture

[CR12] Beavis, S., Beavis, F., & Somerville, P. W. S. A. (2005). Spatial and temporal variability of acidity at a coastal acid sulfate soil site II: A time for change. *Regolith 2005: Ten years of CRC LEME—Proceedings of the CRC LEME regional regolith symposia*, 22–26. Retrieved from http://crcleme.org.au/Pubs/Monographs/regolith2005/Beavis_S_&_Beavis_F_et_al.pdf

[CR13] Beavis, S., Somerville, P., Isaacson, L., Kehoe, M., Beavis, F. R., Kirstie, D., & Welch, S. A. (2006). Sources, sinks, and fluxes of acidity in a coastal acid sulfate soils site. *Goldschimdt Conference 2006*, *70*(18), A43. 10.1016/j.gca.2006.06.195

[CR14] Beavis, S. (2005). Sustainable education in water resources management—Linking undergraduate learning, research and community needs. In: *29th hydrology and water resources symposium: Water capital, 20–23 February 2005, Rydges Lakeside, Canberra*. Retrieved from https://search.informit.com.au/documentSummary;dn=977199898193669;res=IELENG

[CR15] Boto, K. G., & Bunt, J. S. (1981). Dissolved Australian oxygen and pH relationships mangrove waterways in northern. Limnology and Oceanography, 26(6), 1176–1178. Retrieved from https://aslopubs.onlinelibrary.wiley.com/doi/epdf/10.4319/lo.1981.26.6.1176

[CR16] Carlson RR, Foo SA, Asner GP (2019). Land use impacts on coral reef health: A ridge-to-reef perspective. Frontiers in Marine Science.

[CR17] Carpenter, C., & Lawedrau, A. (2002). Effects of forestry activities on surface water quality in the Pacific region : A case study of the Rewa River catchment, Fiji Islands. *The International Forestry Review*, *4*(4), 307–309.

[CR18] Chapman, D. (1996). Water quality assessments—A guide to use of Biota, sediments and water in environmental monitoring—Second Edition. Retrieved from https://apps.who.int/iris/bitstream/handle/10665/41850/0419216006_eng.pdf?se

[CR19] Christie, P., Stevenson, T., Pietri, D., & Field Coordinator, P. (2011.). Lessons from the US Coral triangle initiative support program photo credit: Patrick Christie. Retrieved from http://coraltriangleinitiative.org/sites/default/files/resources/LP%20report_FINAL.pdf

[CR20] Comber, A., & Wulder, M. (2019). Considering spatiotemporal processes in big data analysis : Insights from remote sensing of land cover and land use. 879–891. 10.1111/tgis.12559

[CR21] Comeros-Raynal MT, Lawrence A, Sudek M, Vaeoso M, McGuire K, Regis J, Houk P (2019). Applying a ridge-to-reef framework to support watershed, water quality, and community-based fisheries management in American Samoa. Coral Reefs.

[CR22] Cooke, S. E., & Prepas, E. E. (1998). Stream phosphorus and nitrogen export from agricultural and forested watersheds on the Boreal Plain. Canadian Journal of Fisheries and Aquatic Sciences, 55(10), 2292–2299. 10.1139/f98-118

[CR23] CTI-CFF. (2017). Coral Triangle Initiative. Latin NCAP Activities Report 2017. (November), 28.

[CR24] Dadhich A, Ankita P, Dadhich AP, Nadaoka K (2020). Analysis of terrestrial discharge from agricultural watersheds and its impact on nearshore and offshore reefs in Fiji analysis of terrestrial discharge from agricultural watersheds and its impact on nearshore and offshore reefs in Fiji. Journal of Coastal Research.

[CR25] Delevaux, J. M. S., Whittier, R., Stamoulis, K. A., Bremer, L. L., Jupiter, S., Friedlander, A. M., & Ticktin, T. (2018). A linked land-sea modeling framework to inform ridge-to-reef management in high oceanic islands. In *PLoS ONE* (Vol. 13). 10.1371/journal.pone.0193230 10.1371/journal.pone.0193230 PMC5851582 29538392

[CR26] Destandau, F., & Zaiter, Y. (2020). Spatio-temporal design for a water quality monitoring network maximizing the economic value of information to optimize the detection of accidental pollution. *Water Resources and Economics*. 10.1016/j.wre.2020.100156

[CR27] Dewan, A. M., & Yamaguchi, Y. (2009). Using Remote Sensing and GIS to Detect and Monitor Land Use and Land Cover Change in Dhaka Metropolitan of Bangladesh during, *1960–2005*, 237–249. 10.1007/s10661-008-0226-5 10.1007/s10661-008-0226-5 18317939

[CR28] Dodds, W. K., Justin N., Murdock, M. J., Bernot, R., & Bernot, J. O. (2006). Freshwater ecology: Laboratory manual. *Kansas State University Division of Biology*, (BIOL 612), 1–38. Retrieved from http://citeseerx.ist.psu.edu/viewdoc/download?doi=10.1.1.134.6098&rep=rep1&type=pdf

[CR29] Druschke CG (2013). Watershed as common-place: Communicating for conservation at the watershed scale. Environmental Communication.

[CR30] Duarte CM, Hendriks IE, Moore TS, Olsen YS, Steckbauer A, Ramajo L, McCulloch M (2013). Is ocean acidification an open-ocean syndrome? Understanding anthropogenic impacts on seawater pH. Estuaries and Coasts.

[CR31] EFL. (2012). Nadarivatu hydroelectric scheme official opening Friday 14th September, 2012 project factsheet Nadarivatu hydroelectric scheme official opening. Retrieved from http://efl.com.fj/wp-content/uploads/2013/12/Nadarivatu-Hydro-Scheme-Fact-Sheet.pdf

[CR32] Earth Engine. (2020). Google earth engine data catalogue. Retrieved October 12, 2019, from GEE website: https://code.earthengine.google.com/

[CR33] Ellison, J. C. (2010). Vulnerability of Fiji’s mangroves and associated coral reefs to climate change. A review. In *Awsassets.Panda.Org*. Retrieved from http://awsassets.panda.org/downloads/review_of_fiji_s_mangroves_web_version.pdf

[CR34] ERP. (2019). The Fiji REDD+ National Steering Committee Annex. Retrieved from 10.3389/fmars.2019.00562

[CR35] EPA. (2019). January 2019. Retrieved from https://www.epa.gov/sites/production/files/2019-03/documents/basins4.5coremanual.2019.03.pdf

[CR36] FAO. (2020). Crops and Livestock Products Fiji.

[CR37] FAO. (2016). *Country profile – Fiji*. Retrieved from http://www.fao.org/3/ca0385en/CA0385EN.pdf

[CR38] FAO. (2016). Republic of Fiji. In *IMF Staff Country Reports* (Vol. 16). 10.5089/9781513569147.002

[CR39] FAO. (2011). Highlands and Drylands. Retrieved from http://www.fao.org/3/i2248e/i2248e00.pdf

[CR40] FAO. (2005). *Review of the Forest Revenue System*. Retrieved from http://www.fao.org/3/a-af168e.pdf

[CR41] FSC. (2020). *Fiji sugar corporation annual report 2020*. Retrieved from https://d586e4fd-75a4-4863-b488-df5d313b6679.filesusr.com/ugd/660c5d_1a1131915c0e4808b49fee1a00ee4cd7.pdf

[CR42] FSC. (2020). *Tropical cyclone Sarai*. Fiji Sugar Corporation Media Release. Retrieved from https://d586e4fd-75a4-4863-b488-df5d313b6679.filesusr.com/ugd/660c5d_c870ac9374e040fb96327e6bdccff21b.pdf

[CR43] Fagan, L. L., & Anderson, E. (1995). *Water quality in the Ba River and estuary :* Suva.

[CR44] Falkland, T., Vision, F., Action, T. O., Sustainable, T., Management, W., & The, I. N. (2002). From Vision to action -Towards sustainable water management in the Pacific, Theme 1 Overview Report Water Resources Management, Pacific Regional Consultation on Water in Small Island Countries. In *From vision to action—Towards sustainable water management in the Pacific, Theme 1 Overview Report Water Resources Management, Pacific Regional Consultation on Water in Small Island Countries,*. Retrieved from https://www.pacificwater.org/userfiles/file/Overview%20Paper%20THEME%201.pdf

[CR45] Fidelman, P., Evans, L., Fabinyi, M., Foale, S., Cinner, J., & Rosen, F. (2012). Governing large-scale marine commons: Contextual challenges in the Coral Triangle. *Marine Policy*, *36*(1), 42–53. Retrieved from 10.1016/j.marpol.2011.03.007

[CR46] Fiji Bureau of Statistics (2017). 2017 Population and housing census—release 3. In *Fiji Census* (Vol. 11). Retrieved from https://www.m-culture.go.th/mculture_th/download/king9/Glossary_about_HM_King_Bhumibol_Adulyadej’s_Funeral.pdf

[CR47] Ferese, J., Kenny, G., de Wet, N., Limalevu, L., Bhusan, J., & Ratukalou, I. (2000). Climate Change Vulnerability and Adaptation Assessment for Fiji. Retrieved from https://researchcommons.waikato.ac.nz/handle/10289/1569

[CR48] Foale, S., Adhuri, D., Aliño, P., Allison, E. H., Andrew, N., Cohen, P., & Weeratunge, N. (2013). Food security and the Coral Triangle Initiative. *Marine Policy*, *38*, 174–183. Retrieved from 10.1016/j.marpol.2012.05.033

[CR49] Gale, I. (1991). *Hydrogeological map of Viti Levu*. Retrieved from https://catalogue.nla.gov.au/Record/6294251

[CR50] Gillison, A. N., Management, B., & Services, E. (2014). *Grasslands of the Southwest Pacific.* (November). Retrieved from https://www.researchgate.net/profile/Andrew_Gillison/publication/264237158_Grasslands_of_the_Southwest_Pacific/links/5467c32c0cf20dedafcf51ad/Grasslands-of-the-Southwest-Pacific.pdf

[CR51] Global Environment Facility. (2018a). *GEF Pacific ridge to reef programme third meeting of the regional steering committee*. 1–59. Retrieved from https://info.undp.org/docs/pdc/Documents/FJI/GEF-R2R-RSC1-REPORT%20-%2015_03_2017.pdf

[CR52] Global Environment Facility. (2018b). A ridge-to-reef approach for the integrated management of marine, coastal and terrestrial ecosystems in the Seychelles. Retrieved from Global Environment Facility (GEF). https://www.thegef.org/project/ridge-reef-approach-integrated-management-marine-coastal-and-terrestrial-ecosystems

[CR53] Global Environment Facility. (2016). *From ridge to reef 2016*. 95 pages. Retrieved from https://www.thegef.org/sites/default/files/publications/GEF_RidgetoReef2015_r2_Final.pdf

[CR54] Global Environment Facility. (2013). R2R- Pacific islands ridge-to-reef national priorities—Integrated water, land, forest and coastal management to preserve biodiversity, ecosystem services, store carbon, improve climate resilience and sustain livelihoods. Retrieved from Global Environment Facility (GEF). Retrieved from https://www.thegef.org/project/r2r-pacific-islands-ridge-reef-national-priorities-integrated-water-land-forest-and-coastal

[CR55] Global Environment Facility. (2009). Integrated coastal zone management. Retrieved from Global Environment Facility (GEF). Retrieved from https://www.thegef.org/project/integrated-coastal-zone-management

[CR56] Global Environment Facility. (2007). Irrigation and watershed management. Retrieved May 10, 2021, from Global Environment Facility (GEF). Retrieved from https://www.thegef.org/project/irrigation-and-watershed-management

[CR57] Global Environment Facility. (2006). Community-based watershed management project. Retrieved from Global Environment Facility (GEF). Retrieved from https://www.thegef.org/project/community-based-watershed-management-project

[CR58] Global Environment Facility. (2005). Integrated coastal resources management project. Retrieved from Global Environment Facility (GEF). Retrieved from https://www.thegef.org/project/integrated-coastal-resources-management-project

[CR59] Global Environment Facility. (1999a). Integrated watershed management program for the Pantanal and Upper Paraguay River Basin. Retrieved from Global Environment Facility (GEF) https://www.thegef.org/project/integrated-watershed-management-program-pantanal-and-upper-paraguay-river-basin

[CR60] Global Environment Facility. (1999b). Integrated coastal management project. Retrieved from Global Environment Facility (GEF) https://www.thegef.org/project/integrated-coastal-management-project

[CR61] Green RH, Vascotto GL (1978). A method for the analysis of environmental factors controlling patterns of species composition in aquatic communities. Water Research.

[CR62] Greenberg, A. E., Clesceri, L. S., & Eaton, A. D. (1995). Standard methods for the examination of water and wastewater. Retrieved from https://trove.nla.gov.au/work/16646325?q&sort=holdings%20desc&_=1585610344461&versionId=45704677

[CR63] Gunkel G, Kosmol J, Sobral M, Rohn H (2007). Sugar cane industry as a source of water pollution—Case study on the situation in Ipojuca River, Pernambuco, Brazil. Water, Air and Soil Pollution.

[CR64] Haapio O, M., Filho, W. L., Gonzalez, R., Holland, E., & Wairiu, M. (2014). Mapping the economic costs and benefits of Coral Triangle Initiative (CTI) and Mangrove Rehabilitation Projects (MRP) in Solomon Islands: A study of two MPAs and one MRP. International Journal of Sustainable Development and World Ecology.

[CR65] Halse SA, Cale DJ, Jasinska EJ, Shiel RJ (2002). Monitoring change in aquatic invertebrate biodiversity: Sample size, faunal elements and analytical methods. Aquatic Ecology.

[CR66] Hamid A, Bhat SU, Jehangir A (2020). Local determinants influencing stream water quality. Applied Water Science.

[CR67] Hawes, E., & Smith, M. (2005). Riparian Buffer Zones: Functions and Recommended Widths Prepared by. Retrieved from http://eightmileriver.org/resources/digital_library/appendicies/09c3_RiparianBufferScience_YALE.pdf

[CR68] Hietala, J., Vuori, A., Johnsson, P., Pollari, I., Reutemann, W., & Kieczka, H. (2016). Formic acid. *Ullmann’s Encyclopedia of Industrial Chemistry,**1*, 1–22. https://hero.epa.gov/hero/index.cfm/reference/details/reference_id/5016735

[CR69] Hilty, J., Worboys, G., Keeley, A., Woodley, S., Lausche, B., Locke, H., & Tabor, G. (2020). Guidance for conserving connectivity through ecological networks and corridors. *Best Practice Protected Area Guidelines Series No. 30*, (30), 140. Retrieved from https://www.researchgate.net/publication/342749223_Guidelines_for_conserving_connectivity_through_ecological_networks_and_corridors

[CR70] Hewavitharane, C. A., Pickering, T. D., Ciro, R., & Mochioka, N. (2018). Species composition, abundance and seasonal recruitment patterns of freshwater eels (Anguilla spp.) to Viti Levu, Fiji Islands, in the western South Pacific. Marine and Freshwater Research, 69(11), 1704–1711. 10.1071/MF18105

[CR71] Hofmann, G. E., Smith, J. E., Johnson, K. S., Send, U., Levin, L. A., Micheli, F., & Martz, T. R. (2011). High-frequency dynamics of ocean pH: A multi-ecosystem comparison. *PLoS One*, *6*(12). 10.1371/journal.pone.0028983 10.1371/journal.pone.0028983 PMC3242773 22205986

[CR72] Holland, E., von Schuckmann, K., Monier, M., Legeais, J.-F., Prado, S., Sathyendranath, S., & Dupouy, C. (2019). The use of Copernicus MarineService products to describe the State of the Tropical Western Pacific Oceanaround the Islands: A case study In: Copernicus Marine Service Ocean StateReport, Issue 3. *Journal of Operational Oceanography*, *12:sup1*(September), s26–s30. 10.1080/1755876X.2019.1633075

[CR73] Hoorman, J., Hone, T., & Jr, T. S. (2008). Agricultural Impacts on Lake and Stream Water Quality in Grand Lake St . Marys , Western Ohio. Water Air Soil Pollution, 309–322. 10.1007/s11270-008-9692-1

[CR74] Janine, S. (2010). Environment officials monitor mills. *Fiji Sun*. Retrieved from https://fijisun.com.fj/2010/01/09/environment-officials-monitor-mills/

[CR75] Kaiser BA (2014). Watershed Conservation in the Long Run. Ecosystems.

[CR76] Katafono, R. (2017). The commonwealth pacific small states: The future in the mirror of the past. In *A Sustainable Future for Small States: Pacific 2050*. Retrieved from https://books.google.com.au/books?id=zlxoDwAAQBAJ&lpg=PA18&dq=Fiji%20ratifies%20SDGS%20and%202030%20agenda&lr&pg=PA18#v=onepage&q=Fiji%20ratifies%20SDGS%20and%202030%20agenda&f=false

[CR77] Kelly, R. P., Erickson, A. L., & Mease, L. A. (2014). How not to fall off a cliff, or, using tipping points to improve environmental management. *Ecology Law Quarterly*, *41*(4), 843–886. 10.15779/Z38FP1H

[CR78] Keppel, G., & Tuiwawa, M. V. (2010). *Dry zone forests of Fiji : Species composition, life history traits , and conservation*. *8643*. 10.1080/00288250709509738

[CR79] Khan, M. M., Yang, Y., & Din, I. (2019). Impacts of sugar mill’s effluent on soil and plant’s seed germination, Punjab, Pakistan. *Earth Sciences and Environmental Studies*, 623–637. 10.25177/JESES.4.3.RA.507

[CR80] Kirschke, S., Avellán, T., Bärlund, I., Bogardi, J. J., Chapman, D., Dickens, C. W. S., Warner, S. (2020). *Capacity challenges in water quality monitoring : understanding the role of human development*. Retrieved from https://link.springer.com/article/10.1007/s10661-020-8224-3 10.1007/s10661-020-8224-3 PMC7167377 32307607

[CR81] Kolhe, A.S. Sarode, A.G. and, & S.R.Ingale. (2008). Study of Effluent From Sugar Cane Industry. International Research Journal of Sodh, Samiksha & Mulyankan, 4(1), 303–306. Retrieved from https://www.researchgate.net/profile/Ajaykumar-Kolhe/publication/343006972_STUDY_OF_EFFLUENT_FROM_SUGAR_CANE_INDUSTRY/links/5f11546f4585151299a1397d/STUDY-OF-EFFLUENT-FROM-SUGAR-CANE-INDUSTRY.pdf

[CR82] Kuglerová L, Ågren A, Jansson R, Laudon H (2014). Towards optimizing riparian buffer zones: Ecological and biogeochemical implications for forest management. Forest Ecology and Management.

[CR83] Kumar V (2010). Water management in Fiji. International Journal of Water Resources Development.

[CR84] Kumar RS, Swamy RN, Ramakrishnan K (2001). Pollution studies on sugar mill effluent-physico-chemical characteristics and toxic metals. Pollution Research.

[CR85] Kumar, A. (2005). *Geology, Climate, and Landscape of the PABITRA Wet- Zone Transect , Viti Levu Island , Fiji*. *59*(2), 141–157. 10.1353/psc.2005.0018.full

[CR86] Kwong NK, Bholah A, Volcy L, Pynee K (2002). Nitrogen and phosphorus transport by surface runoff from a silty clay loam soil under sugarcane in the humid tropical environment of Mauritius. Agriculture, Ecosystems and Environment.

[CR87] Lagabrielle, Y., Auzende, J., Eissen, J., & Janin, M. (1994). *Geology and geochemistry of a 800 m section through young upper oceanic crust in the North Fiji Basin (Southwest Pacific)*. *116*. Retrieved from https://www.sciencedirect.com/science/article/pii/0025322794901724

[CR88] Lamb, D. (2011). *World Forests—Volume VIII Regreening the Bare Hills*. Retrieved from https://books.google.com.au/books?id=USq4DmDOOH4C&lpg=PR3&dq=FAO%20watershed%20highland%20agriculture%20asia-pacific&lr&pg=PP1#v=onepage&q&f=false

[CR89] Larsen, L. (1999). GIS in environmental monitoring and assessment. *Geographical Information Systems*, 999–1007. Retrieved from http://www.geos.ed.ac.uk/~gisteac/gis_book_abridged/files/ch71.pdf

[CR90] Ledua, E., Matoto, S. V, Sesewa, A., & Korovulavula, J. (1996). *Freshwater Clam Resources of Ba*. Retrieved from https://spccfpstore1.blob.core.windows.net/digitallibrary-docs/files/11/11ee77fcb6cfcde508ef458e22cffd10.pdf?sv=2015-12-11&sr=b&sig=D4kUcrlZkvPbLoRXKjVKedWmO9MX7MxJhoedS%2Bjy1cQ%3D&se=2021-03-07T09%3A36%3A10Z&sp=r&rscc=public%2C%20max-age%3D864000%2C%20max-stale%3D86400&rsct=application%2Fpdf&rscd=inline%3B%20filename%3D%22Ledua_96_Mussels_Fiji.pdf%22

[CR91] Lenat, D. R. (1984). Agriculture and Stream Water Quality: a Biological Evaluation of Erosion Control Practices. Environmental Management, (1980). Retrieved from https://link.springer.com/article/10.1007/BF01868032

[CR92] Li Y, Sun M, Evans KS, Ren Y, Chen Y (2020). Rethinking marine conservation strategies to minimize socio-economic costs in a dynamic perspective. Biological Conservation.

[CR93] MacHiwal D, Jha MK, Mal BC (2011). GIS-based assessment and characterization of groundwater quality in a hard-rock hilly terrain of Western India. Environmental Monitoring and Assessment.

[CR94] Manabe, S., Bryan, K., & Spelman, M. (1990). Transient response of a global ocean-atmosphere model to a doubling of atmospheric carbon dioxide. *Journal of Physical Oceanography*, *20*, 722–749

[CR95] Mander, Ü., Hayakawa, Y., & Kuusemets, V. (2005). Purification processes, ecological functions, planning and design of riparian buffer zones in agricultural watersheds. Ecological Engineering. Retrieved from: https://www.sciencedirect.com/science/article/abs/pii/S0925857405000157

[CR96] Mataki M, Kosby KC, Lal M (2006). Baseline climatology of Viti Levu (Fiji) and current climatic trends. Pacific Science.

[CR97] Matkar LS, Gangotri MS (2002). Physico-chemical analysis of sugar industrial effluents. Journal of Industrial Pollution Control.

[CR98] McCauley DJ, Arnold WJ, Saxton JB, Turner CJ (2019). Applying adaptive management and lessons learned from national assessments to address logistical challenges in the National Wetland Condition Assessment. Environmental Monitoring and Assessment.

[CR99] Mcphie, J. (1994). *A Pliocene shoaling basaltic seamount : Ba Volcanic Group at Rakiraki, Fiji*. *64*, 193–210. Retrieved from https://www.sciencedirect.com/science/article/pii/037702739400050Q

[CR100] Ministry of Lands, Government of Fiji. (2019). Vanua GIS. Retrieved from https://vanuagis.lands.gov.fj/vanuagis/

[CR101] Moritz, C., Vii, J., Lee Long, W., Jerker, T., Thomassin, A., & Planes, S. (2018). Status and trends of coral reefs of the Pacific. 215. Retrieved from https://www.sprep.org/sites/default/files/documents/publications/status-coral-reefs-pacific.pdf

[CR102] Morris, C.-W. (2007). SOUTH-WEST PACIFIC STATUS OF CORAL REEFS. Retrieved from https://www.cbd.int/doc/meetings/mar/rwebsa-wspac-01/other/rwebsa-wspac-01-fiji-coral-reefs-en.pdf

[CR103] Morrison, G., Fatoki, O. S., Persson, L., & Ekberg, A. (2001). Assessment of the impact of point source pollution from the Keiskammahoek Sewage Treatment Plant on the Keiskamma River—pH , electrical conductivity , oxygen- demanding substance (COD) and nutrients. *Water South Africa*, *27*(4), 475–480. Retrieved from https://www.ajol.info/index.php/wsa/article/view/4960

[CR104] NASA. (2009). South-West Pacific Status of Coral Reefs. (January).

[CR105] NASA. (2015). Exploring reefs from space. *Exploring Reefs from Space*. Retrieved from https://earthobservatory.nasa.gov/images/86163/exploring-reefs-from-space

[CR106] Naidu PN, Khan MGM, Jokhan AD (2017). Assessment of sugarcane varieties for their stability and yield potential in Fiji. The South Pacific Journal of Natural and Applied Sciences.

[CR107] Neumann, M., & Dudgeon, D. (2002). The impact of agricultural runoff on stream benthos in Hong Kong. 36, 3103–3109. Retrieved from https://reader.elsevier.com/reader/sd/pii/S0043135401005401?token=E82D77A3BACE401C43BD317D6948E54E50B454EE1217DB986EBA7B58D576DEB5CE9907495E2AA95C560B992EC8692115 10.1016/s0043-1354(01)00540-1 12171409

[CR108] Naidu, S. (1988). *Water quality in the Monosavu Reservoir and Wailoa River in 1987*. Retrieved from http://uspaquatic.library.usp.ac.fj/gsdl/collect/kalenchits_m-moanausp/index/assoc/HASHbeef.dir/doc.pdf

[CR109] Naidu, S., & Brodie, J. E. (1987). *Water quality in the Monosavu Reservoir and Wailoa River in 1986*. Retrieved from http://uspaquatic.library.usp.ac.fj/gsdl/collect/kalenchits_m-moanausp/index/assoc/HASHef6e.dir/doc.pdf

[CR110] Nhiwatiwa T, Dalu T, Brendonck L (2017). Impact of irrigation based sugarcane cultivation on the Chiredzi and Runde Rivers quality, Zimbabwe. Science of the Total Environment.

[CR111] Norton, S. A., B. J. Cosby, I. J. Fernandez, J. S. Kahl, AND M R. Church. . EGS, (2001). LONG-TERM AND SEASONAL VARIATIONS IN CO2; LINKAGES TO CATCHMENT ALKALINITY GENERATION. HYDROLOGY AND EARTH SYSTEM SCIENCES, 5(1), 83–91. Retrieved from https://cfpub.epa.gov/si/si_public_record_report.cfm?Lab=NHEERL&count=10000&dirEntryId=83989&searchall=&showcriteria=2&simplesearch=0&timstype=

[CR112] Observatory of Economic Complexity. (2020). Fiji Trade Overview. Retrieved from https://oec.world/en/profile/country/fji

[CR113] Olajire AA, Imeokparia FE (2001). Water quality assessment of osun river: Studies on inorganic nutrients. Environmental Monitoring and Assessment.

[CR114] Ourbak T, Magnan AK (2018). The Paris Agreement and climate change negotiations: Small islands, big players. Regional Environmental Change.

[CR115] Ovalle, A. R. G., Rezende, C. E., Lacerda, L. D., & Silva, C. A. R. (1990). Factors Affecting the Hydrochemistry of a Mangrove Tidal Creek, Sepetiba Bay , de daneiro State t tudy area Sepetiba Bay Creek mouth Study area low mangrove ~ Mud flat A-J Sampling points. Estuarine, Coastal and Shelf Science, 639–650. Retrieved from https://www.sciencedirect.com/science/article/abs/pii/027277149090017L

[CR116] Paris, A., Veirus, T., & Gehrig, S. (2019). Fiji Ridge to Reef Project Activity 1.1.1.2: Vanua o Votua Shark and Ray Survey Report August 2019.

[CR117] Pietri, D. M., Stevenson, T. C., & Christie, P. (2015). The Coral Triangle Initiative and regional exchanges: Strengthening capacity through a regional learning network. Global Environmental Change, 33, 165–176. 10.1016/j.gloenvcha.2015.05.005

[CR118] Printemps, J. (2008). *Integrated coastal management—GERSA project spatial approach—Remote sensing mapping potential erosion risks in North Viti Levu ((Fiji)Fiji) using the USLE model and a GIS author :* Retrieved from https://spccfpstore1.blob.core.windows.net/digitallibrary-docs/files/ad/ad2c3d6a3725333662bb7b4f0691854e.pdf?sv=2015-12-11&sr=b&sig=R%2FpeeK89kW7ypL7RXas3lvKZfOhUDgdJbO7ZBroWWco%3D&se=2020-12-28T14%3A08%3A11Z&sp=r&rscc=public%2C%20max-age%3D864000%2C%20max-stale%3D86400&rsct=application%2Fpdf&rscd=inline%3B%20filename%3D%22ENG_2008_Erosion_risks_Viti_Levu.pdf%22

[CR119] Peterjohn, W. T., & Correll, D. T. (1984). The effect of riparian forest on the volume and chemical composition of baseflow in an agricultural watershed. Smithsonian Environmental Research Center. Retrieved from https://repository.si.edu/bitstream/handle/10088/17779/serc_Peterjohn_Correll_1986_WtsdPerspectives.pdf

[CR120] Qamese, S. (2018). Combining observations with GR4J catchment model to understand water quality and the impacts of climate change in the upper Ba Catchment of Viti Levu, Fiji by Semi Qamese. Doctorate dissertation (March).

[CR121] Ram, A. R. (2013). Rainfall, runoff events and fluvial sediment delivery patterns in small forested coastal watersheds in Southern Viti Levu, Fiji Islands by (University of the South Pacific). Retrieved from http://digilib.library.usp.ac.fj/gsdl/collect/usplibr1/index/assoc/HASH01bc/671932fb.dir/doc.pdf

[CR122] Rashni. B. (2020). Session 2, Topic 2 freshwater invertebrate assemblages and ecological status of the Ba River, Fiji by Bindiya Rashni 1. (2020). (February), 1–24. Retrieved from https://www.pacific-r2r.org/sites/default/files/2020-03/RSTC-TC-SI-WP.%207_Session%202%20-%20Topic%202%20Freshwater%20Invertebrate%20Assemblages%20and%20Ecological%20Status%20of%20the%20Ba%20River%2C%20Fiji%20%28revised%29.pdf

[CR123] Reefbase. (2020). ReefGIS. Retrieved from http://www.reefbase.org/gis_maps/

[CR124] Rodda, P. (1966). Geology of Viti Levu, Fiji/geology compiled by P. Rodda and R. B. Band ; drawn by R. Narayan ; topography from Directorate of Overseas Surveys. Retrieved from https://nla.gov.au/nla.obj-540309433/view

[CR125] SDSN. (2021). 14.3 minimize and address the impacts of ocean acidification, including through enhanced scientific cooperation at all levels. Retrieved from SDSN website website: https://indicators.report/targets/14-3/

[CR126] SPREP. (2007). *Sustainable integrated water resources and countries national integrated water resource management diagnostic report Fiji Islands table of contents*. Retrieved from https://www.sprep.org/att/IRC/eCOPIES/Countries/Fiji/49.pdf

[CR127] Sahu O (2018). Assessment of sugarcane industry: Suitability for production, consumption, and utilization. Annals of Agrarian Science.

[CR128] Sahu, O. P., & Chaudhari, P. K. (2015). The characteristics, effects, and treatment of wastewater in the sugarcane industry. *Water Quality, Exposure and Health*, *7*(3), 435–444. https://link.springer.com/article/10.1007/s12403-015-0158-6

[CR129] Samuel, S., & Muthukkaruppan, S. M. (2011). Physico-chemical analysis of sugar mill effluent, contaminated soil and its effect on seed germination of paddy (Oryza sativa L.). *International Journal of Pharmaceutical & Biological Archives*, *2*(5), 1469–1472. http://citeseerx.ist.psu.edu/viewdoc/download?doi=10.1.1.860.5918&rep=rep1&type=pdf

[CR130] Santos IR, Glud RN, Maher D, Erler D, Eyre BD (2011). Diel coral reef acidification driven by porewater advection in permeable carbonate sands, Heron Island Great Barrier Reef. Geophysical Research Letters.

[CR131] Silaitoga, S. (2009). Dirty mill wins more time. *Fiji Times*, pp. 1–2. Retrieved from https://search-proquest-com.virtual.anu.edu.au/docview/376885336?pq-origsite=summon

[CR132] Soldatenko SA, Yusupov RM (2019). Estimating the influence of thermal inertia and feedbacks in the atmosphere-ocean system on the variability of the global surface air temperature. Izvestiya - Atmospheric and Ocean Physics.

[CR133] Stephens, M., Lowry, J. H., & Ram, A. R. (2018). Location-based environmental factors contributing to rainfall-triggered debris flows in the Ba river catchment, northwest Viti Levu island, Fiji. (November 2017), 145–159. 10.1007/s10346-017-0918-4

[CR134] Stewart-oaten, A. (1996). Goals in environmental monitoring. In *Detecting ecological impacts. Concepts and applications in coastal habifals, e*. Retrieved from https://www.sciencedirect.com/science/article/pii/B9780126272550500045

[CR135] Stevenson, R. J., Bennett, B. J., Jordan, D. N., & French, R. D. (2012). Phosphorus regulates stream injury by filamentous green algae , DO , and pH with thresholds in responses. 25–42. 10.1007/s10750-012-1118-9

[CR136] SPC. (2016). Pacific Ridge to Reef Programme REPORT First meeting of the Regional Steering Committee and Inception Workshop. Retrieved from: https://info.undp.org/docs/pdc/Documents/FJI/GEF-R2R-RSC1-REPORT%20-%2015_03_2017.pdf

[CR137] SPC. (2018). GEF Pacific Ridge to Reef Programme Third Meeting of the Regional Steering Committee. 1–59. Retrieved from https://info.undp.org/docs/pdc/Documents/FJI/GEF-R2R-RSC1-REPORT-15_03_2017.pdf

[CR138] SPC. (2020). Regional International Waters R2R Project. Retrieved from Web page website: https://www.pacific-r2r.org/regional-international-waters-r2r-project

[CR139] Taba, Y., Osborne, T., Rounds, I., Strand, E., Sevutia, S., Taylor, J. E. Niukula, J. (2005). Invasive-plant assessment and weed management plan for the Fijian Crested Iguana Sanctuary Island this report was compiled by. (August).

[CR140] Tamata, B. R., & Kubuabola, S. (1993). *Bale R. Tamata, M.Env.Stud. Sereana Kubuabola, MSc.* Suva.

[CR141] Terry JP, Lal R, Garimella S (2008). An examination of vertical accretion of floodplain sediments in the labasa river sugarcane belt of northern Fiji: Rates, influences and contributing. Geographical Research.

[CR142] Terry, J. P. (2001). Rapid rates of channel migration in a Pacific island river. *Journal of Pacific Studies*, (January). Retrieved from https://www.researchgate.net/profile/James_Terry5/publication/257144931_Rapid_rates_of_channel_migration_in_a_Pacific_island_river/links/5433ab4d0cf20c6211be6068/Rapid-rates-of-channel-migration-in-a-Pacific-island-river.pdf

[CR143] Terry, J. P., Garimella, S., & Kostaschuk, R. A. (2002). Rates of floodplain accretion in a tropical island river system impacted by cyclones and large floods. *42*, 171–182. Retrieved from https://reader.elsevier.com/reader/sd/pii/S0169555X01000848?token=D3311E1E27216AD8FF68695B15ECDBEE16AD639ABA4A48A775A4703011FA98D48DA9FEB735BD176614D58DB1E7159430

[CR144] Tibby, J., Reid, M. A., Fluin, J., Hart, B. T., & Kershaw, A. P. (2003). Assessing long-term pH change in an Australian river catchment using monitoring and palaeolimnological data. Environmental Science & Technology, 37(15), 3250–3255. https://pubs.acs.org/doi/abs/10.1021/es0263644 10.1021/es0263644 12966966

[CR145] Tucker, C. C., & Robinson, E. H. (1990). Channel catfish farming handbook. Springer Science & Business Media. Retrieved from: https://books.google.com.au/books?hl=en&lr=&id=PdxLfZah5V0C&oi=fnd&pg=PR9&dq=affect+stream+pH+either+making+it+too+acidic+or+alkaline+(Robinson,+1990).&ots=NSsVtCyxw-&sig=P4fc9UU27preiLDahWU34NxC2rM

[CR146] Tuivanualevu, F. (2017). How does the law protect rivers in Fiji from pollution? Retrieved from SAS Ocean Law Bulletins website: http://www.sas.com.fj/ocean-law-bulletins/how-does-the-law-protect-rivers-in-fiji-from-pollution

[CR147] Tuiwawa, S. H., Skelton, P. A., & Tuiwawa, M. (2013). A field guide to the species of mangroves and sea - grasses of the Fiji Islands. Retrieved from A field guide to the species of mangroves and sea - grasses of the Fiji Islands.

[CR148] Tuqiri, N. K. (2009). Reef fish assessment in Fiji’s *“i-qoliqoli.”* Retrieved from https://www.grocentre.is/static/gro/publication/213/document/nanise09prf.pdf

[CR149] Turinayo, Y. K. (2016). Impact of wastewater effluents from a sugar industry and a molasses based distillery on water*.* (Makarere University). 10.13140/RG.2.2.30789.45280

[CR150] Turner, A. M., & Trexler, J. C. (1997). Sampling aquatic invertebrates from marshes : Evaluating the options author (s): Andrew M . Turner and Joel C. Trexler Published by : The University of Chicago Press on behalf of the Society for Freshwater Science Stable. *16*(3), 694–709. https://www.jstor.org/sta

[CR151] UN Sustainable Development Solutions Network (UNSDSN). (2020). Indicators and a monitoring framework launching a data revolution for the sustainable development goals: Indicators by target. Retrieved April 12, 2020, from https://indicators.report/targets/

[CR152] UNDP. (2020). Fiji Ridge to Reef Project. Retrieved from https://www.pacific.undp.org/content/pacific/en/home/projects/fiji-r2r.html

[CR153] Revised to: UNEP-WCMC, WorldFish Centre, WRI, TNC (2021). Global distribution of warm-water coral reefs, compiled from multiple sources including the Millennium Coral Reef Mapping Project. Version 4.1. Includes contributions from IMaRS-USF and IRD (2005), IMaRS-USF (2005) and Spalding et al. (2001). Cambridge (UK): UN Environment World Conservation Monitoring Centre. Data DOI: https://doi.org/10.34892/t2wk-5t34

[CR154] USGS (2020). USGS Earth Explorer. Retrieved from Earth Explorer website: https://earthexplorer.usgs.gov/

[CR155] Van Metre, P. C., Qi, S., Deacon, J., Dieter, C., Driscoll, J. M., Fienen, M., & Wolock, D. (2020). Prioritizing river basins for intensive monitoring and assessment by the US Geological Survey. *Environmental Monitoring and Assessment*, *192*(7). 10.1007/s10661-020-08403-1 10.1007/s10661-020-08403-1 PMC7320945 32594332

[CR156] Veitayaki J (1998). Traditional and community-based marine resources management system in Fiji: An evolving integrated process. Coastal Management.

[CR157] Veitayaki, J., Waqalevu, V., Varea, R., & Rollings, N. (2017). Participatory mangrove management in a changing climate. In *Participatory Mangrove management in a changing climate*. 10.1007/978-4-431-56481-2_19

[CR158] Vierus T, Gehrig S, Brunnschweiler JM, Zimmer M, Marie AD, Rico C (2018). Discovery of a multispecies shark aggregation and parturition area in the Ba Estuary. Fiji Islands (april).

[CR159] Vieux BE (2016). Distributed Hydrologic Modeling. Encyclopedia of GIS.

[CR160] von Schuckmann, K., Le Traon, P. Y., Smith, N., Pascual, A., Djavidnia, S., Gattuso, J. P., … Zunino, S. (2020). Copernicus Marine Service Ocean State Report, Issue 4. Journal of Operational Oceanography, 13(S1), S1–S172. 10.1080/1755876X.2020.1785097

[CR161] Vuki, V., Naqasima, M., & Vave, R. (2000). *Status of Fiji’s coral reefs*. Retrieved from http://www.reefbase.org/resource_center/publication/main.aspx?refid=10975

[CR162] WWF. (2003). *S*etting priorities for marine conservation in the Fiji Islands marine ecoregion. Retrieved from https://earthobservatory.nasa.gov/images/86163/exploring-reefs-from-space

[CR163] Wilson, M. (2015). Pacific Ridge ‐ to ‐ Reef Program : Status Update Rationale of GEF Funded Intervention. (October). Retrieved from https://www.thegef.org/sites/default/files/events/Pacific_Ridge-to-Reef_Program_ECW_MAW_Update_Oct_15.pdf

[CR164] Wooldridge SA (2009). Water quality and coral bleaching thresholds: Formalising the linkage for the inshore reefs of the Great Barrier Reef, Australia. Marine Pollution Bulletin.

[CR165] Yeo, S. W., Blong, R. J., Mcaneney, K. J., Yeo, S. W., Blong, R. J. (2007). *Flooding in Fiji : Findings from a 100-year historical series*. *6667*. 10.1623/hysj.52.5.1004

[CR166] Yeo, S., & Yeo, S. W. (2016). *Ba community flood preparedness project: Final report Ba Community flood preparedness project: Final Report. Department of Physical Geography*. (April 2000).

[CR167] Yu K, DeLaune RD, Tao R, Beine RL (2008). Nonpoint source of nutrients and herbicides Associated with sugarcane production and its impact on Louisiana coastal water quality. Journal of Environmental Quality.

[CR168] Zed, P. (1987). Acacias in Fiji. In *Australian acacias in developing countries* (pp. 4–7). Retrieved from https://ageconsearch.umn.edu/record/134369/files/PR016.pdf#page=183

[CR169] Zeng Z, Estes L, Ziegler AD, Chen A, Searchinger T, Hua F, Wood F, E. (2018). Highland cropland expansion and forest loss in Southeast Asia in the twenty-first century. Nature Geoscience.

[CR170] Zhang Y, Chen Z, Zhu B, Luo X, Guan Y, Guo S, Nie Y (2008). Land desertification monitoring and assessment in Yulin of Northwest China using remote sensing and geographic information systems (GIS). Environmental Monitoring and Assessment.

[CR171] Zimmerman, S., & Kazandijan, V. (2003). *Statistical quality control using excel*. Retrieved from https://dl.acm.org/doi/book/10.5555/862479

[CR172] Zinabu, E., Kelderman, P., van der Kwast, J., & Irvine, K. (2019). Monitoring river water and sediments within a changing Ethiopian catchment to support sustainable development. *Environmental Monitoring and Assessment*, *191*(7). 10.1007/s10661-019-7545-6 10.1007/s10661-019-7545-6 PMC6588641 31227917

[CR173] Zhou, Y., Zhao, B., Peng, Y., & Chen, G. (2010). Influence of mangrove reforestation on heavy metal accumulation and speciation in intertidal sediments. Marine Pollution Bulletin, 60(8), 1319–1324. 10.1016/j.marpolbul.2010.03.010 10.1016/j.marpolbul.2010.03.010 20378130

